# Collagen XII Plays a More Prominent Cell‐Mediated Role in Tendon Organization Compared to Matrix Assembly During Postnatal Development

**DOI:** 10.1096/fj.202501618R

**Published:** 2025-10-29

**Authors:** Ashley K. Fung, Stephanie N. Weiss, Courtney A. Nuss, William Yen, Susan W. Volk, Nathaniel A. Dyment, Louis J. Soslowsky

**Affiliations:** ^1^ McKay Orthopaedic Research Laboratory, Department of Orthopaedic Surgery University of Pennsylvania Philadelphia Pennsylvania USA; ^2^ Department of Bioengineering University of Pennsylvania Philadelphia Pennsylvania USA; ^3^ Department of Clinical Sciences and Advanced Medicine School of Veterinary Medicine, University of Pennsylvania Philadelphia Pennsylvania USA

**Keywords:** cell organization, collagen XII, extracellular matrix, mechanics, tendon

## Abstract

Tendon structural and mechanical integrity is essential for overall joint function. Establishment of tendon structure–function is regulated by coordinated processes between cells and the extracellular matrix. However, the cell–cell and cell–matrix interactions necessary for re‐creating the native tendon structure following injury remain unelucidated. Collagen XII is a fibril‐associated collagen with key structural roles in modulating collagen fibrillogenesis, matrix protein interactions, and forming bridges between fibrils. Collagen XII has also been shown to regulate cell structure and organization, suggesting that collagen XII coordinates cell‐ and matrix‐mediated processes necessary for proper tendon formation. Therefore, the study objective was to define the temporal roles of collagen XII in regulating cell arrangement and matrix assembly during tendon development. To investigate the cumulative effects of manipulating collagen XII expression in tendon with respect to cell organization, scleraxis‐Cre collagen XII knockout mice were evaluated for patellar tendon morphology, cell organization, matrix structure and function, and gene expression. At postnatal day 0, disruptions to cell and matrix organization due to collagen XII knockout were profound. F‐actin organization in knockout tendons lacked parallelism with areas of high density, and cell membrane protrusions did not make contact with neighboring cells. Disrupted matrix structure was also observed at later postnatal ages, indicating a critical cell‐mediated role of collagen XII. Collagen XII knockout also inhibited the formation of a proper tibial attachment, resulting in altered collagen organization and mechanical properties at the insertion site. To investigate the specific matrix assembly role of collagen XII, knockdown was induced at postnatal day 0. Mechanical changes at the tibial insertion site were similar, but overall effects on matrix organization and structure were minimal. Together, our findings indicate a more substantial role of collagen XII for regulating cell arrangement rather than matrix assembly in the establishment of tendon structure–function.

## Graphical abstract

Temporal roles of collagen XII in regulating cell and matrix organization during tendon development were investigated by tendon‐targeted knockout throughout development and induced knockdown at birth. Tendon‐targeted knockout disrupted patellar tendon morphology, cell and matrix organization, and mechanical properties. When knockdown was induced at birth, mechanical changes were similar, but effects on cell and matrix organization were minimal. Our findings indicate a more substantial role for collagen XII in regulating cell arrangement rather than matrix assembly in tendon development.

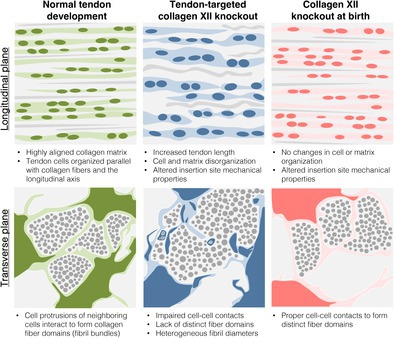

## 1 Introduction

The highly organized, hierarchical structure of tendon directly influences mechanical function and efficient force transmission between muscle and bone [1, 2]. Though matrix proteins are the key components that give tendons their strength, structural features of tendon fibroblasts are also essential during collagen fibrillogenesis and matrix assembly. Fibroblasts provide extracellular compartmentalization due to the unique architecture of their surface. Fibripositors have been hypothesized to dictate the parallelism of fibrils during fibrillogenesis [1], while fibril bundles influence how collagen fibers branch, bifurcate, and rotate along the tendon axis [3]. As lateral growth progresses, cell protrusions regulate how adjacent fibers form fascicles. Though the microenvironments defined by the fibroblast surface are crucial for spatially regulating fibrillogenesis and higher‐order matrix assembly [4], the coordinated cell–cell and cell–matrix mechanisms underlying the development of tendon hierarchical structure are not fully understood, and their re‐establishment following injury remains a significant clinical challenge.

Collagen XII is a fibril‐associated collagen with interrupted triple helices (FACIT). Collagen XII binds to collagen I via its collagenous domains, and its unique, large, third non‐collagenous region extends into the interfibrillar space. The fibril‐associated nature of collagen XII implicates it in both the processes of fibril formation and modulating interactions between fibrils, cells, and other extracellular matrix (ECM) components. For example, the long non‐collagenous domain interacts with other matrix molecules, such as tenascin‐X and decorin [5, 6], to form flexible bridges between collagen fibrils and modulate fibril organization. Clinically, mutations in the *Col12a1* gene result in a mixed myopathic and Ehlers‐Danlos syndrome (EDS), termed myopathic EDS. Patients present with symptoms such as, distal joint hypermobility, proximal joint contractures, abnormal scarring, muscle weakness, and an absence of deep tendon reflexes [7]. A *Col12a1* global knockout mouse model recapitulates some of the key neuromuscular syndromes observed in myopathic EDS patients, such as a reduction in grip strength [8]. Flexor digitorum longus (FDL) tendons also demonstrated abnormalities in fiber and fibril structure, where postnatal day 30 global knockout mice exhibited poorly defined fiber domains, disrupted organization, and truncated cellular processes [9]. Tendon findings in the global *Col12a1* knockout model support key roles for collagen XII in regulating fibrillogenesis, fiber and fibril organization, as well as a more novel role in regulating cell shape and organization. However, a limitation of this model is the potential confounding effects of collagen XII knockout in other tissues, such as muscle and bone.

To specifically isolate the role of collagen XII in tendon, we previously developed and characterized a conditional *Col12a1* ^flox/flox^ mouse model crossed with scleraxis (Scx)‐Cre mice to obtain tendon‐targeted collagen XII knockout (denoted as ScxCre‐KO) mice [10]. In mature (day 60) mice, ScxCre‐KO FDL and patellar tendons were less stiff, and patellar tendons showed a significant reduction in dynamic modulus with a corresponding increase in phase shift. The degree to which collagen fibers realign with loading was also diminished in KO tendons, together indicating alterations in both viscoelasticity and matrix organization. However, given the additional emerging role of collagen XII in cell organization, it remains unclear whether the disrupted tendon matrix structure and function are driven by disordered cellular arrangement early in development or by altered fibrillogenesis in the extracellular space.

Therefore, our overall objective was to define the temporal roles of collagen XII in regulating the establishment of tendon structure–function during development. We hypothesized that collagen XII regulates tendon cell organization during early development, prior to significant extracellular matrix deposition, which is pivotal for establishing normal structure–function. To test this hypothesis, our objectives were to define the temporal roles of collagen XII in regulating (1) cell arrangement and (2) matrix assembly during tendon development. Two mouse models were used to delineate the initial role of collagen XII in cell arrangement from that in matrix assembly. Tendon‐targeted scleraxis‐Cre collagen XII knockout (ScxCre‐KO) mice were used to knockout collagen XII throughout tendon development, while conditional *Col12a1* mice were crossed with Rosa‐CreER^T2^ mice to generate ubiquitous tamoxifen‐inducible collagen XII knockout (RosaCre‐KO) mice. Tamoxifen was administered to newborn pups on postnatal days 0 and 1 to induce knockdown following the establishment of cell organization. Patellar tendons were assessed at postnatal days (*p*) 0, 10, and 30.

## 2 Methods

### 2.1 Study Design

All animal work was approved by the University of Pennsylvania Institutional Animal Care and Use Committee. A multidisciplinary approach including cell morphology and organization as well as matrix structure and function analyses was conducted. Cell organization assays included cell morphology and organization via whole mount confocal microscopy (*n* = 4–6/group) and transverse transmission electron microscopy (TEM, *n* = 4–6/group), and gene expression analysis of target genes (*n* = 4–10/group). Matrix structure assays included tendon morphology via paraffin histology (*n* = 4–8/group), whole mount second harmonic generation (SHG) imaging (*n* = 4–6/group) and fibril morphology (*n* = 4–6/group). Finally, matrix function was assessed with quasi‐static and viscoelastic mechanical analyses (*n* = 12/group). An approximately equal number of male and female mice was used for all ages. Mechanical assessment was not conducted for p0 tendons, and at p30, both sexes were evaluated (*n* = 12/group). For all other p30 assays, results were stratified when there were sex differences. All assays were conducted in a randomized order by a blinded investigator.

### 2.2 Mouse Models

Tendon‐targeted scleraxis‐Cre; *Col12a1* ^flox/flox^ collagen XII knockout (ScxCre‐KO) mice were used. Scx‐Cre mice were bred with *Col12a1* ^flox/flox^ mice to homozygosity, resulting in a final breeding scheme of female Scx‐Cre+; *Col12a1* ^flox/flox^ mice crossed with male *Col12a1* ^flox/flox^ mice. Female Cre+ mice were used exclusively for breeding to eliminate the possible confounding factor of Cre recombination in the sperm of male mice. Cre− littermates were used as controls. Rosa‐CreER^T2^ mice were crossed with *Col12a1* ^flox/flox^ conditional mice to homozygosity to generate Rosa‐CreER^T2+/−^;*Col12a1* ^flox/flox^ breeders. The final experimental breeding scheme was Rosa‐CreER^T2+/−^;*Col12a1* ^flox/flox^ mice crossed with *Col12a1* ^flox/flox^ mice, and Cre− littermates were used as controls. At postnatal days 0 and 1, tamoxifen was injected subcutaneously at a dose of 100 mg/kg bodyweight. Tamoxifen was dissolved in corn oil at a concentration of 5 mg/mL, and a 20 μL dose was administered for a 1 g pup. To account for any off‐target effects, experimental and control pups received tamoxifen. And to avoid potential recombination or downstream effects of tamoxifen in breeders, female mice were sacrificed after their first experimental litter, and male mice were removed from the cage prior to administration of tamoxifen.

### 2.3 Tendon Morphology

At time of harvest, knees from p0, p10, and p30 mice were dissected, positioned at 90° within histology cassettes, and fixed in 4% paraformaldehyde for 1 day at 4°C. Knees from p10 and p30 mice were decalcified in a solution of 0.5 M ethylenediaminetetraacetic acid (EDTA) and 1% paraformaldehyde at 4°C for 7–10 days with switches to fresh solution every 2–3 days. Samples were then processed through an ethanol series, infiltrated with paraffin, and embedded. Patellar tendons were sectioned at a 7 μm thickness in the sagittal plane, and sections were stained with 0.1% toluidine blue and imaged at 20× magnification using a ZEISS Axioscan 7. Patellar tendon length was measured using ImageJ by averaging the distance from the patella to the tibial insertion sites on both the anterior and posterior edges of the tendon. Genotypes within each age were compared using unpaired, two‐tailed, Student's *t*‐tests. Significance was set at *p ≤* 0.05.

### 2.4 Cell and Matrix Organization

Whole patellar tendons were harvested, stained, and optically cleared prior to SHG imaging and confocal microscopy to visualize F‐actin, nuclei, and the collagen matrix. At the time of harvest, knees from p0, p10, and p30 mice were dissected, positioned at 90° within histology cassettes, and fixed in 4% paraformaldehyde at 4°C. After 2–4 h of fixation, patellar tendons were dissected from the joint and placed back into 4% paraformaldehyde overnight. Patellar tendons were then blocked and permeabilized for a day (10% bovine serum albumin, 1% Triton‐X), incubated in Alexa Fluor 555+ phalloidin (1:500, ThermoFisher A30106) and DRAQ5 (1:1000, ThermoFisher 62252) for a day, rinsed with phosphate‐buffered saline, and optically cleared using increasing fructose concentrations (20%–115% wt/vol) [11]. Tendons were incubated at 4°C until they were transferred to 80% fructose at room temperature. Samples were mounted in slides with concavities, and z‐stacks were acquired (30–50 μm thickness) using a multiphoton microscope to visualize collagen (SHG), F‐actin, and nuclei. Collagen density was calculated as an average of the forward and backward SHG channels, and the OrientationJ plug‐in on ImageJ was used to quantify matrix organization. Matrix organization was quantified using circular standard deviation, where lower values are indicative of more aligned collagen. Maximum projections of z‐stack subsets ranging from 2 to 5 μm thick depending on age were evaluated for nuclear morphology and cell density. CellProfiler and custom MATLAB scripts were used to segment nuclei and calculate nuclear aspect ratio and orientation. Within each age, collagen density, matrix organization, cell density, and nuclear organization were compared between genotypes using unpaired, two‐tailed, Student's *t*‐tests while nuclear aspect ratio distributions were compared using Kolmogorov–Smirnov tests. Significance was set at *p ≤* 0.05.

### 2.5 Collagen Fibril Structure

Patellar tendons were harvested from p0, p10, and p30 mice for evaluation of fibril structure via transmission electron microscopy. For p0 and p10 mice, whole knees were immersed in fixative, while for p30, patellar tendons were fixed in situ for 5 min, dissected out of the joint, and placed in fixative. Tendons were fixed in Karnovsky's fixative (4% paraformaldehyde, 2.5% glutaraldehyde, 0.1 M sodium cacodylate, 8.0 mM calcium chloride) for 2–4 h at 4°C. Following fixation, samples were rinsed in 0.1 M sodium cacodylate buffer. Patellar tendons from p0 and p10 mice were dissected from the knee joints, and p30 tendons were cleaned to remove excess surrounding tissue. Tendons were then post‐fixed in 1% osmium tetroxide for an hour at 4°C, dehydrated in a series of increasing ethanol concentrations at room temperature, and infiltrated with propylene oxide prior to embedding in Epon at 60°C. Blocks were sectioned in the transverse plane of the tendon, and sections (50–100 nm thick) were post‐stained with UranyLess (EMS 22409) and 1% phototungstic acid. A transmission electron microscope (JEOL 1010) was used to image sections at magnifications ranging from 10000 to 60000× to visualize cell, fiber, and fibril structure. Collagen fibril diameters were measured from images acquired at 60000× using a custom MATLAB script. Within each age, fibril diameter distributions were compared between genotypes using Kolmogorov–Smirnov tests. Significance was set at *p ≤* 0.05.

### 2.6 Viscoelastic Mechanical Testing

Patellar tendon‐tibial complexes from p10 and p30 mice were dissected and cleaned of surrounding musculature. Stain lines were applied using Verhoeff's stain for optical strain tracking, cross‐sectional area was measured using a custom laser device [12], and tibias were secured in polymethylmethacrylate. Custom fixtures were used to grip the patella. For p10 tendons, the patella was sandwiched between sandpaper using cyanoacrylate glue, and for p30 tendons, a custom‐built hook‐like fixture was used to support the bottom edge of the patella for tensile testing. Tendons were loaded into a mechanical testing system (5848, Instron, Norwood, MA) in a phosphate buffer saline bath maintained at 37°C. Patellar tendons were preloaded to 0.05 N, and the testing protocol consisted of preconditioning, a stress relaxation test at 6% strain followed by a dynamic frequency sweep (0.1, 1, 5, 10 Hz), and finally a quasi‐static ramp to failure at a rate of 0.1% strain/s. Images were acquired during the quasi‐static ramp using a quantitative polarized light imaging (QPLI) set‐up to calculate collagen fiber organization [13, 14, 15, 16]. Variance in angle of polarization was determined during loading, where a lower value is indicative of more aligned collagen. Within each age and sex (for p30 mice), genotypes were compared using unpaired, two‐tailed, Student's *t*‐tests. Significance was set at *p ≤* 0.05.

### 2.7 Gene Expression

Immediately following euthanasia, knee joints and patellar tendons were harvested for gene expression analysis. Due to the difficulty of quickly and confidently dissecting tendons from p0 mice, p0 patellar tendons were assessed via a fixation and microdissection‐based method. Knee joints from p0 mice were fixed in 4% paraformaldehyde for 3 h at 4°C and then embedded in optical cutting temperature (OCT) compound. Sagittal cryosections were acquired at 40 μm thickness, and a 25G needle was used to dissect and isolate the patellar tendon from each section. The tendon was digested with proteinase K, and RNA was isolated (Quick‐RNA Microprep, Zymo) as described [17]. For p10 and p30 mice, patellar tendons were dissected at the patellar and tibial insertions to encompass the entirety of the tendon and subsequently stored in RNAlater (AM7021, ThermoFisher). At the time of RNA extraction, tendons were mechanically homogenized using a pestle in a phenol‐based lysis reagent (QIAzol, Qiagen 79306), phase‐separated using chloroform, and RNA was isolated according to manufacturer's protocols (Direct‐zol RNA Microprep, Zymo, R2062). RNA was converted to cDNA (4374966, ThermoFisher) and pre‐amplified for 12 cycles (100‐5581, Standard BioTools, San Francisco, CA) for 94 target genes (*Rn18s* and *Col1a1* were excluded from preamplification due to high expression). Pre‐amplified cDNA was then loaded into a 96.96 Dynamic Array IFC (BMK‐M‐96.96, Standard BioTools, San Francisco, CA). Target genes included those related to collagens, non‐collagenous matrix proteins, matrix remodeling, cell–cell and cell–ECM interactions, signaling pathways, and cell markers (Table S1). Δ*Ct* values were calculated by normalization to the average *Ct* value of the housekeeper genes, *Abl1* and *Rps17*. Principal component analysis between genotypes was conducted using custom MATLAB code. Within each age, genotypes were compared using unpaired, two‐tailed, Student's *t*‐tests. Significance was set at *p ≤* 0.05. Raw data is included in the supplemental data (Table S2).

### 2.8 Western Blots

Immediately following euthanasia, patellar tendons were dissected at the patella and tibial insertion sites and flash frozen in liquid nitrogen. Tendons were later homogenized in RIPA buffer (9806, Cell Signaling) with protease inhibitor (1:100, P8340, Sigma‐Aldrich), phosphatase inhibitor cocktail 2 (1:100, P5726, Sigma‐Aldrich), and phosphatase inhibitor cocktail 3 (1:100, P0044, Sigma‐Aldrich) using a bead beater homogenizer with ceramic beads (2.8 mm, 15‐340‐154, ThermoFisher). The lysate was collected and vortexed, and the supernatant was collected. A BCA assay (Pierce 23227) was used to determine protein concentrations. 1.5 μg of protein was then boiled at 95°C in 1X SDS lysis buffer (NP0007, Invitrogen) with 5% beta mercaptoethanol. Proteins were separated on 3%–8% tris acetate gels (EA0375, Invitrogen) in tris acetate run buffer (LA0041, Invitrogen) and transferred onto PVDF membranes in transfer buffer (NP00061, Invitrogen) containing 10% methanol. Blots were blocked in 5% milk in TBS containing 0.1% Tween‐20, incubated in collagen XII (1:3000, rabbit anti‐collagen XII KR33, gift from Dr. Manuel Koch, University of Cologne) and β‐actin (1:500, Cell Signaling 4967) primary antibodies, followed by incubation in secondary antibodies (Rabbit HRP, Cell Signaling 7074, 1:4000–1:2000). Quantification by densitometry was performed using ImageJ, and collagen XII relative density was normalized to β‐actin relative density.

### 2.9 Patellar Tendon Tibial Attachment Histology

At time of harvest, knees from p30 mice were dissected, positioned at 90°, and fixed in 4% paraformaldehyde for 1 day at 4°C. Knees were then rinsed and submerged in 30% sucrose for a day at 4°C prior to embedding in optical cutting temperature (OCT) compound. Sagittal plane sections were acquired at both 7 μm thickness for mineral staining and 50 μm thickness to visualize collagen via SHG imaging. Sections for mineral assessment were stained with calcein blue (M1255, Sigma‐Aldrich) and alkaline phosphatase (E6588, ThermoFisher) as described [18], and imaged at 10× magnification using a ZEISS Axioscan 7. Sections for collagen assessment were stained with DRAQ5 (1:1000, ThermoFisher 62252) and imaged at 40× magnification on a multiphoton microscope (Leica TCS SP8 MP).

## 3 Results

### 3.1 Establishment of *Col12a1* Knockdown in ScxCre‐KO Line

The cumulative effects of manipulating collagen XII expression in tendon with respect to cell organization as well as structural and mechanical properties were analyzed in patellar tendons of tendon‐targeted ScxCre;*Col12a1* ^flox/flox^ (ScxCre‐KO) mice at postnatal days (p) p0, p10, and p30. At each age, ScxCre‐KO mice were compared to their respective Cre‐ littermate controls. These postnatal ages correspond with key developmental timepoints. At p0, cell organization into linear arrays with cell extensions has been established, but the extracellular matrix is still immature [19]. By p10, tendon growth shifts from cell proliferation to matrix production and assembly [20]. And by p30, the hierarchical cell and matrix structure has been established, but mice have not yet reached sexual maturity.

**FIGURE 1:**
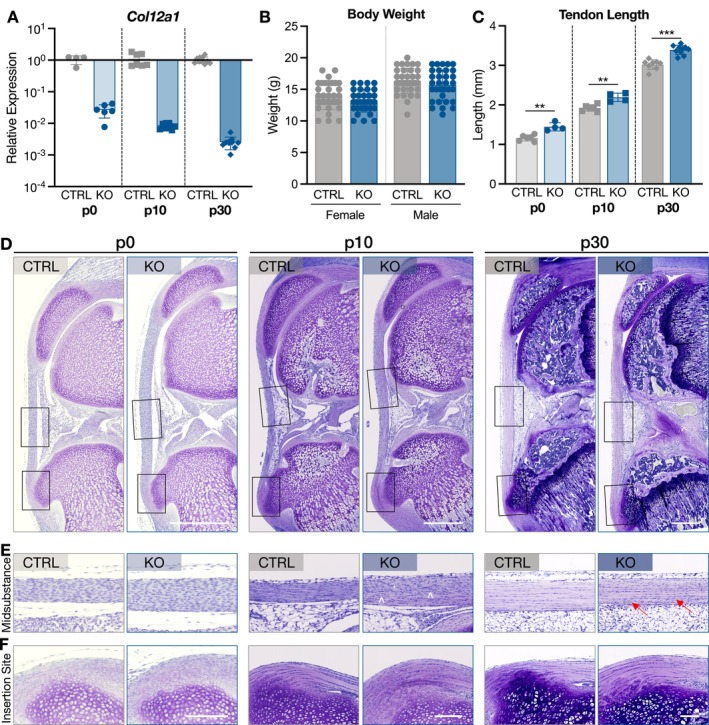
Collagen XII regulates patellar tendon length and development of the tibial attachment. (A) *Col12a1* gene expression was substantially reduced in ScxCre‐KO mice compared to CTRL. ScxCre‐KO expression is relative to respective CTRL expression within each age. (B) Body weights between CTRL and ScxCre‐KO mice were not different for female or male p30 mice. Weights for all p30 mice used are presented. (C) ScxCre‐KO patellar tendons were longer at all ages. Data presented as mean ± standard deviation (**p < 0.01, ***p < 0.001). (D) Representative images of patellar tendons. Scale bar = 200 μm. (E) Regional evaluation of the midsubstance and insertion site demonstrated that cells were disorganized in p10 ScxCre‐KO tendons (^), while p30 ScxCre‐KO tendons had areas of higher cell density compared to CTRL (red arrows). Additionally, formation of the tibial attachment was diminished in ScxCre‐KO tendons at all ages. Scale bar = 50 μm.

Consistent with mature mice and as expected, *Col12a1* gene expression was substantially reduced in ScxCre‐KO patellar tendons at all ages (Figure 1A). Additionally, though previous work showed that ScxCre‐KO mice were smaller in day 60 mice compared to littermate controls (CTRL) [10], body weight was not different in either female or male mice at p30 (Figure 1B).

### 3.2 Collagen XII Regulates Patellar Tendon Length and Development of the Tibial Attachment

In previous work, day 60 ScxCre‐KO tendons were longer than CTRL; therefore, p30 tendons were assessed to determine if lengthening occurred by this age. ScxCre‐KO p30 patellar tendons were similarly longer than CTRL tendons, and this finding was consistent at p10 and p0, ages at which matrix assembly is much less established (Figure 1C,D). The differences in patellar tendon length between CTRL and ScxCre‐KO tendons at each age were similar with averages of 0.24 mm, 0.27 mm, and 0.38 mm at p0, p10, and p30, respectively. Therefore, this suggests that the growth rate of CTRL and ScxCre‐KO tendons is consistent postnatally, and the increased length observed at mature ages is not initiated by cumulative effects during postnatal development.

Within the patellar tendon midsubstance, there were no qualitative differences at p0, while p10 KO tendons demonstrated areas of cell disorganization, as indicated by disruptions in the parallelism of cell arrays (Figure 1E). At p30, areas of high cell density were also observed in ScxCre‐KO tendons. Interestingly, collagen XII knockout had a striking effect on the formation of the tibial attachment (Figure 1F). At p10 and p30, the tibial tuberosity of ScxCre‐KO mice was less pronounced, had reduced proteoglycan staining, and the organization was altered with disruptions to the columnar cell structure between tendon and bone observed in CTRL tendons.

### 3.3 Collagen XII Regulates Nuclear Morphology and Cell Structure

**FIGURE 2:**
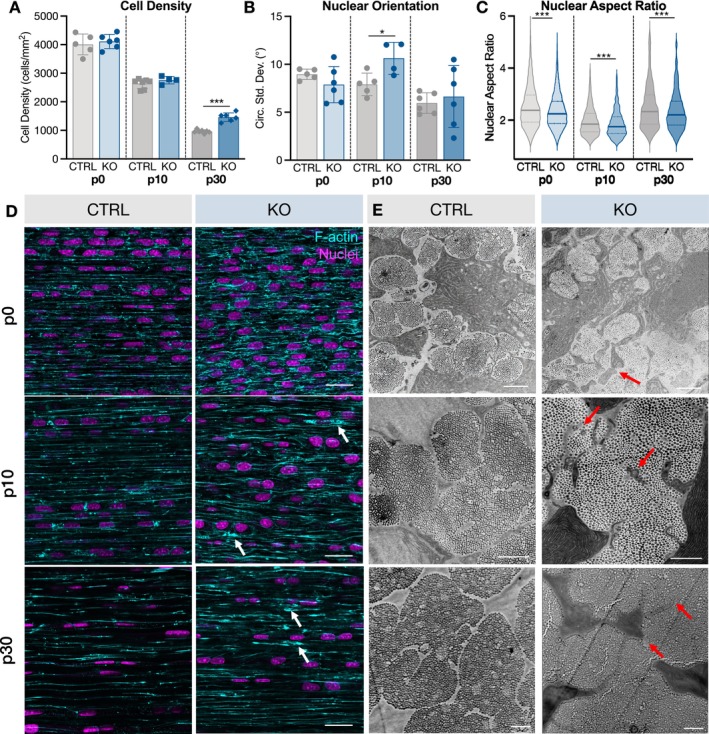
Collagen XII regulates nuclear morphology and cell structure. (A) Cell density was not different between genotypes at p0 or p10 but higher in ScxCre‐KO p30 tendons. (B) Nuclear circular standard deviation was higher in p10 ScxCre‐KO tendons compared to CTRL, indicating nuclear disorganization. Data presented as mean ± standard deviation. (*p < 0.05, ***p < 0.001). (C) Nuclei in ScxCre‐KO tendons were rounder at all ages. (D) Representative nuclei images of CTRL and ScxCre‐KO tendons. Scale bar = 25 μm. CTRL tendons had F‐actin organization with high parallelism, consistent with the longitudinal axis of the tendon. In contrast, F‐actin organization in ScxCre‐KO tendons was disrupted at all ages with lack of parallelism and areas of high density (white arrows). Scale bar = 50 μm. (E) CTRL tendons demonstrated clear fibril bundles with boundaries defined by the cell. In contrast, p0 and p10 ScxCre‐KO tendons had membrane protrusions that failed to make contacts with neighboring cells and an abundance of fibripositors (red arrows). In p30 ScxCre‐KO tendons, cell protrusions did not form connections with neighboring cells. Scale bar = 1 μm.

To further investigate the role of collagen XII in regulating cell organization, whole patellar tendons were stained with phalloidin and DRAQ5 to visualize F‐actin and nuclei, respectively. Altered cell structure was observed early at p0 despite no differences in cell density or nuclear orientation (Figure 2A,B). Nuclei were slightly rounder, and there was a greater density of disordered F‐actin filaments (Figure 2C,D). In contrast, CTRL tendons had F‐actin filaments aligned parallel with the long axis of the tendon. Irregular cell shape in KO tendons was also observed in transverse transmission electron microscopy (TEM) cross‐sections (Figure 2E). In p0 CTRL tendons, cell protrusions interacted with those of neighboring cells showing the emergence of clearly defined fibril bundles. In p0 ScxCre‐KO tendons, however, cell protrusions failed to establish clear fibril bundles. At p10, nuclei in p10 ScxCre‐KO tendons were more disorganized (Figure 2B). Consistent with p0, we observed slightly rounder nuclei, disordered F‐actin filaments, and the absence of clear fiber domains (Figure 2C–E). In normal development, the number of fibripositors typically decreases by p10, but KO tendons had an increased abundance of fibripositors localized around fragmented protrusions (Figures 2E and S1B–G). Finally, at p30, cell density was 52% greater in ScxCre‐KO tendons compared to control (Figure 2A). Though there were no differences in nuclear orientation, nuclei were again slightly rounder (Figure 2B,C). When stratified by sex, the difference in nuclear aspect ratio for p30 tendons was largely driven by more rounded cells in male ScxCre‐KO tendons (Figure S1A). Alterations in F‐actin filament staining were less striking at p30, but there were areas of high density and disordered filaments, and cell protrusions did not form connections with neighboring cells (Figure 2D,E). Altogether, cell organization and structure were impaired as early as p0, with clear effects on the establishment of hierarchical structure.

### 3.4 Collagen Fibril Structure Is Altered, and Matrix Organization Is Disrupted in the Absence of Collagen XII

**FIGURE 3:**
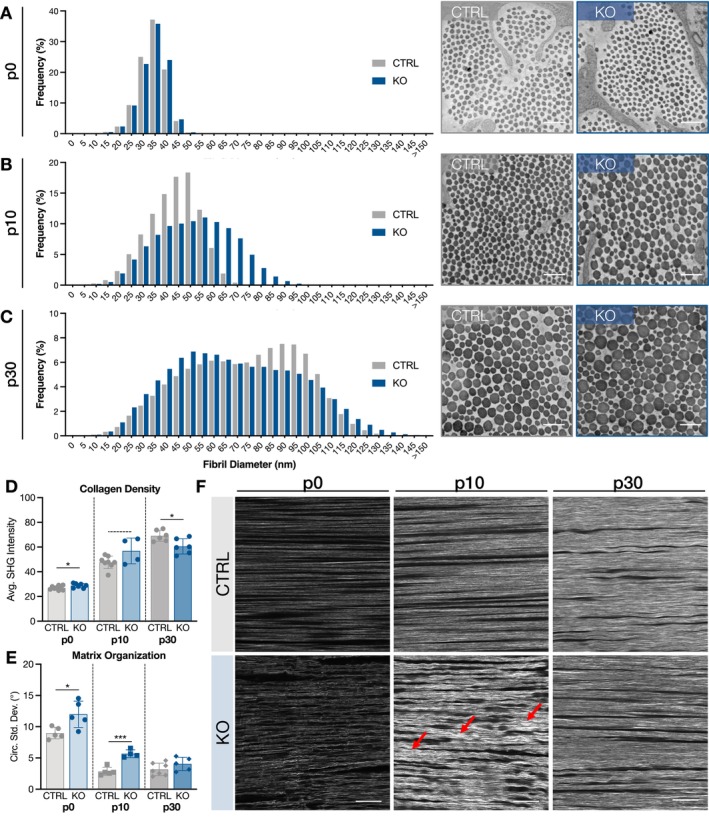
Collagen fibril structure is altered, and matrix organization is disrupted in the absence of collagen XII. (A) Fibril diameter distributions at p0 were not different between genotypes. (B) At p10, there was a substantial shift towards larger fibril diameters in ScxCre‐KO tendons with greater fibril diameter heterogeneity. (C) At p30, the fibril diameter distribution of ScxCre‐KO tendons had a higher peak at smaller fibril diameters, while CTRL tendons had a higher peak at larger fibril diameters. Scale bar = 200 nm. (D) Collagen density was higher in p0 and p10 ScxCre‐KO tendons but lower in p30 ScxCre‐KO tendons compared to CTRL. (E, F) In contrast to the highly aligned matrix observed in CTRL tendons, p0 and p10 ScxCre‐KO tendons exhibited matrix disorganization, as indicated by greater circular standard deviation, with the presence of misaligned and wavy fibers (red arrows). At p30, matrix disorganization of ScxCre‐KO tendons was less severe than younger ages, but areas of wavy fibers persisted. Data presented as mean ± standard deviation (—*p* < 0.1, *p < 0.05, ***p < 0.001). Scale bar = 50 μm.

Collagen fibril structure was evaluated using transmission electron microscopy, and collagen density and matrix alignment in the longitudinal plane were assessed via second harmonic generation (SHG) imaging in the midsubstance region of patellar tendons. At p0, fibril diameter distributions were not substantially different in ScxCre‐KO tendons (Figure 3A), though collagen density was slightly higher (Figure 3D). Strikingly, despite an early stage of matrix deposition, collagen fibers were clearly disorganized at p0 with the presence of misaligned and wavy fibers and increased circular standard deviation (Figure 3E,F). Changes in fibril and fiber structure were more pronounced at p10. The fibril diameter distribution of ScxCre‐KO tendons exhibited greater heterogeneity with a higher percentage of larger diameter fibrils as compared to CTRL (Figure 3B). Overall collagen density was moderately higher at p10 (Figure 3D), and SHG images demonstrated areas of high intensity in KO tendons. Similar to p0, portions of the collagen fiber matrix lacked parallelism with disrupted organization (Figure 3E). The irregularity of the period and amplitude of the wavy fibers indicates that these findings are not simply the appearance of crimp (Figure 3F).

In p30 CTRL tendons, the fibril diameter distribution was bimodal, as expected, with a higher second peak (90 nm) of larger fibril diameters. ScxCre‐KO tendons also exhibited a bimodal distribution, but with a higher first peak (50 nm) of smaller fibril diameters (Figure 3C), suggesting that lateral association is inhibited during growth [21, 22]. Additionally, ScxCre‐KO tendons had a greater percentage of large diameter fibrils (> 115 nm). Collagen density was lower in p30 ScxCre‐KO tendons compared to CTRL (Figure 3D), and though there were still some areas of wavy fibers, matrix alignment did not appear appreciably different from CTRL tendons (Figure 3F).

### 3.5 Collagen XII Regulates Tendon Mechanical Properties

**FIGURE 4:**
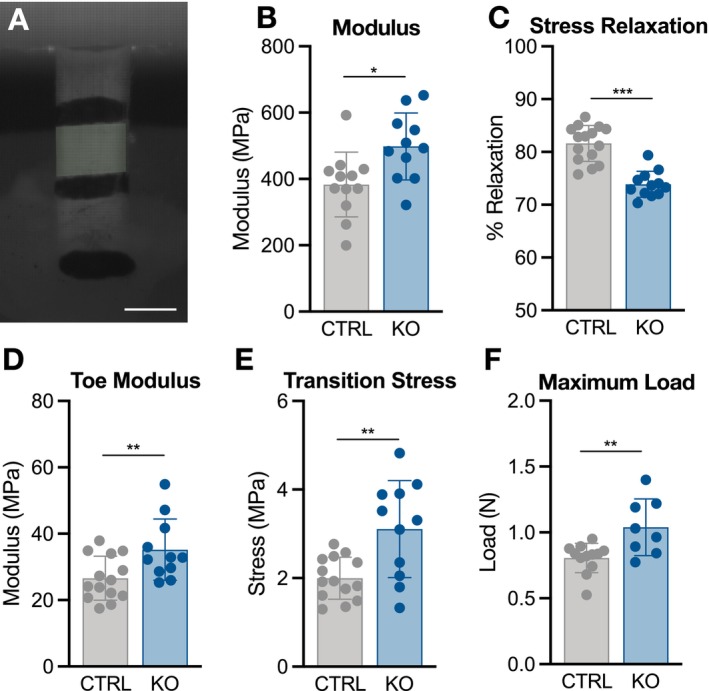
Collagen XII knockout increases tendon elastic and failure mechanical properties at p10. (A) A p10 patellar tendon loaded for mechanical testing. The shaded midsubstance region was optically tracked to calculate modulus. (B) Modulus was higher, while (C) percent relaxation was lower in ScxCre‐KO tendons. (D) Toe modulus, (E) transition stress and (F) maximum load were higher in ScxCre‐KO tendons. Data presented as mean ± standard deviation (*p < 0.05, **p < 0.01, ***p < 0.001).

**FIGURE 5:**
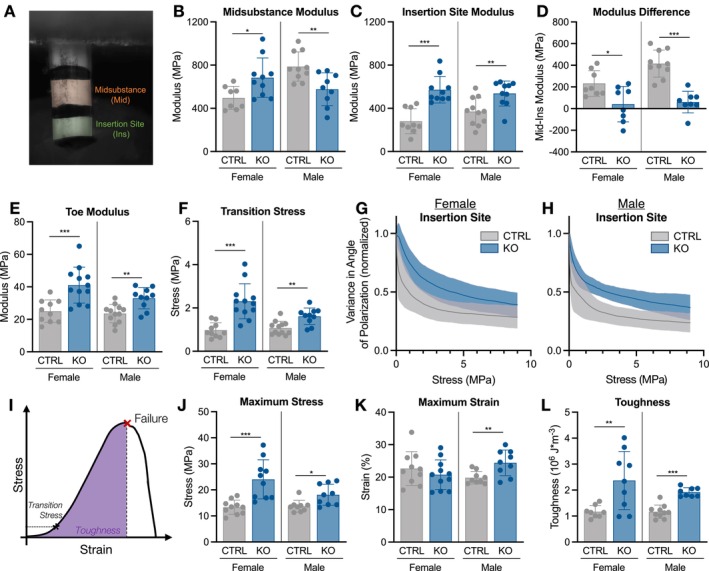
Collagen XII knockout increases tendon insertion site elastic and failure mechanical properties at p30. (A) A p30 patellar tendon loaded for mechanical testing. The shaded insertion site (Ins) and midsubstance (Mid) regions were optically tracked to calculate modulus. (B) Midsubstance modulus was higher in female ScxCre‐KO tendons but lower in males. (C) Insertion site modulus was higher in KO tendons. (D) Modulus difference, calculated as Mid‐Ins modulus, was diminished in ScxCre‐KO tendons. (E) Toe modulus and (F) transition stress were higher in ScxCre‐KO tendons. KO tendons demonstrated reduced collagen fiber realignment in the Ins region of both (G) females and (H) males. Solid lines are the mean with standard deviation represented by the shaded region. (I) Maximum stress, strain, toughness, and toe region properties were calculated. Toughness (purple) was calculated as the area under the stress–strain curve to failure. (J, K) ScxCre‐KO tendons failed at a higher stress, with greater maximum strain in male ScxCre‐KO tendons. (L) Toughness was higher in ScxCre‐KO tendons. Data presented as mean ± standard deviation (*p < 0.05, **p < 0.01, ***p < 0.001).

To assess whether matrix disorganization has functional implications, patellar tendon mechanical properties were evaluated in p10 and p30 mice (Figures 4 and 5). Structurally, cross‐sectional area was not different between groups at p10, while ScxCre‐KO tendons were longer, supporting our histological findings (Figure S2). Though linear stiffness was not different, ScxCre‐KO tendons surprisingly exhibited a higher modulus of the midsubstance, calculated via optical strain tracking of the central midsubstance region (Figures S2 and 4A,B). Viscoelastic analysis, which consisted of a 6% strain stress relaxation test followed by a dynamic frequency sweep, also revealed less stress relaxation in ScxCre‐KO tendons, and a reduced phase shift (a measure of the lag in stress response with applied strain) (Figures 4C and S3).

Given the stark differences in matrix organization as well as the reductions in tendon elastic modulus observed in mature mice [23], the greater elastic mechanical behavior observed in ScxCre‐KO tendons was unexpected. Interestingly, toe modulus was greater in ScxCre‐KO tendons, and this was predominantly driven by a higher transition stress, defined as the point between toe and linear regions (Figure 4D,E). The differences in toe modulus and transition stress point to alterations in matrix organization that impact toe region loading mechanisms, such as fiber uncrimping and sliding. Finally, tendon failure primarily occurred at the tibial insertion site, with ScxCre‐KO tendons failing at a higher load compared to CTRL, but with no difference in maximum strain (Figures 4F and S2). Technical limitations inhibited the ability to measure cross‐sectional area at the insertion site, and therefore, properties specific to this region, such as modulus and maximum stress, cannot be reliably reported.

At p30, mechanical property differences were comparable to those observed at p10 with enhanced findings at the tibial insertion site. Stratification by sex was necessary due to differences observed across several parameters. Stain lines were optically tracked to calculate regional moduli (Figure 5A). Surprisingly, female ScxCre‐KO tendons had a higher midsubstance modulus compared to CTRL, while the modulus was lower in male ScxCre‐KO tendons (Figure 5B). Given the insertion site changes observed histologically, the insertion site was also evaluated, and the modulus of this region was higher in both female and male ScxCre‐KO tendons (Figure 5C). While the sex differences in midsubstance modulus were surprising, when the difference between midsubstance and insertion site modulus was calculated, collagen XII knockout affected female and male tendons similarly with a diminished modulus difference (Figure 5D). Structurally, ScxCre‐KO tendons were smaller only in female mice and longer for both sexes (Figure S4). Male p30 ScxCre‐KO tendons were less stiff than CTRL, but there were no differences in female ScxCre‐KO tendons (Figure S4). In contrast to p10, there were no changes in percent relaxation, while male KO tendons had a lower dynamic modulus across all frequencies, and female KO tendons exhibited a similar reduction in phase shift to p10 (Figures S3 and S4).

Similar to p10, toe modulus was higher in both female and male ScxCre‐KO tendons, and this was again driven by increased transition stress (Figure 5E,F). To ask whether alterations in dynamic loading processes were driving these changes, quantitative polarized light imaging analysis was conducted during the quasi‐static ramp to assess the degree of collagen fiber realignment with loading. Interestingly, throughout the toe region (stress < 4 MPa), significantly less fiber realignment occurred in the insertion site of ScxCre‐KO tendons with only minor differences in the midsubstance (Figures 5G,H and S4). Failure also occurred in this region, with ScxCre‐KO tendons failing at a greater maximum stress (Figure 5I,J). While maximum strain was only higher in male ScxCre‐KO tendons, toughness, or energy to failure, was greater in all ScxCre‐KO tendons (Figure 5K,L). Interestingly, histological evaluation via SHG in p30 patellar tendons showed that, in contrast to CTRL tendons, KO tendons lacked a clear tidemark distinguishing mineralized fibrocartilage, collagen fibers were disorganized, and there was aberrant mineralization within the attachment site (Figure S5). Altogether, our findings suggest that alterations in matrix organization due to the absence of collagen XII alter tendon elasticity, toe region behavior, and failure properties, particularly at the tendon insertion site.

### 3.6 Collagen XII Knockout Alters Tendon Gene Expression Profiles

**FIGURE 6:**
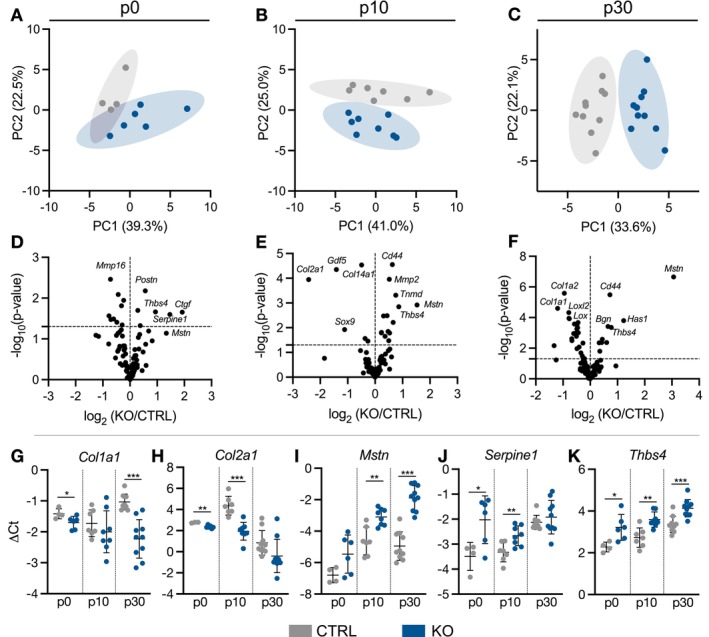
Collagen XII knockout alters tendon gene expression profiles. (A–C) Principal component analysis demonstrated clustering based on genotype at each age. (D–F) Volcano plots show differential gene expression between CTRL and ScxCre‐KO groups for each age. ScxCre‐KO tendons had decreased expression of (G) *Col1a1* and (H) *Col2a1* and increased expression of (I) *Mstn*, (J) *Serpine1*, and (K) *Thbs4*. Data presented as mean ± standard deviation (*p < 0.05, **p < 0.01, ***p < 0.001).

Gene expression was assessed via high‐throughput qPCR for selected target genes (Table S1) related to the categories of collagens, non‐collagen matrix proteins, matrix remodeling, cell–cell and cell–matrix interactions, cell markers, and signaling pathways. We first performed principal component analysis (PCA) at each age. Clusters separated by genotype in the second principal component (PC2) in p0 and p10 tendons (Figure 6A,B) and strengthened to the first (PC1) by p30 (Figure 6C). Volcano plots highlight the magnitude of the gene expression differences (Figure 6D–F). Expression of several genes, such as *Col1a1* (Figure 6G), *Col2a1* (Figure 6H), *Sox9, and Gdf5*, was reduced in ScxCre‐KO tendons, while expression of genes such as *Mstn*, *Serpine1*, and *Thbs4*, was elevated across multiple ages (Figure 6I–K). Notably, *Col1a1*, *Col1a2*, *Lox*, and *Loxl2* had an approximately 2–3‐fold reduction in p30 ScxCre‐KO tendons, while *Mstn* increased 8‐fold. Together, gene expression results suggest altered cell phenotype due to collagen XII knockout.

Altogether, results reveal profound effects of collagen XII knockout on cell and matrix organization, structure, and overall tendon function. Understanding the coordinated cell–cell and cell–matrix interactions that drive the establishment of structure–function remains a significant gap in knowledge. In the context of collagen XII, significant changes in cell structure and F‐actin organization at p0 suggest that disordered cell arrangement leads to disrupted tendon hierarchical structure. Interestingly, the observation of disorganized matrix at this timepoint also suggests a potential role of matrix assembly in altering cell arrangement. Therefore, we next sought to delineate the role of collagen XII in matrix assembly from that in the initial establishment of cell organization.

### 3.7 Induced Collagen XII Knockdown Stunts Growth With Minimal Changes in Tendon Morphology

**FIGURE 7:**
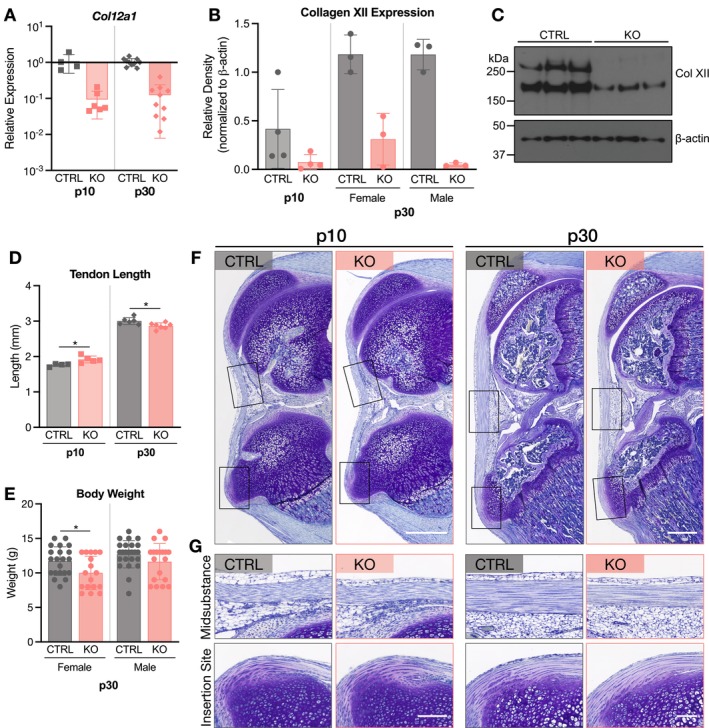
Induced collagen XII knockdown (RosaCre‐KO) stunts growth with minimal changes in tendon morphology. (A–C) *Col12a1* and collagen XII expression was reduced in p10 and p30 RosaCre‐KO tendons. RosaCre‐KO gene expression is relative to the respective CTRL group within each age. (D) At p10, KO tendons were minimally longer, while p30 RosaCre‐KO tendons were shorter than CTRL. (E) Female and male p30 RosaCre‐KO mice were smaller than CTRL. Data is shown for all Rosa p30 mice used. (F) Representative images of p10 and p30 patellar tendons. Scale bar = 200 μm. (G) Regional analysis showed no qualitative differences in the midsubstance or insertion site regions. Scale bar = 50 μm. Data presented as mean ± standard deviation (*p < 0.05, ***p < 0.001).

To test the role of collagen XII in hierarchical matrix assembly after tenocytes have organized into linear arrays, ubiquitous tamoxifen‐inducible Rosa‐CreER^T2^; *Col12a1* ^f/f^ (RosaCre‐KO) mouse models were used and compared to Cre− littermate controls (CTRL). To induce knockdown of collagen XII after cell arrangement has been established, but prior to substantial matrix assembly, tamoxifen was injected subcutaneously at postnatal days 0 and 1. Tendons were then evaluated at p10 and p30. *Col12a1* knockdown efficiency was evaluated in this mouse model, and while the magnitude of knockdown did not reach the same level as that in ScxCre‐KO tendons, *Col12a1* gene and protein expression were still substantially reduced in RosaCre‐KO tendons at both p10 and p30 (Figure 7A–C).

Though tendon length was statistically greater in RosaCre‐KO tendons at p10, the difference was small (Figure 7D). In contrast, RosaCre‐KO tendons were shorter at p30, and this could be attributed to reduced mouse size at this age (Figure 7E). No qualitative differences in morphology were observed in the midsubstance and insertion site regions of the patellar tendon (Figure 7F,G).

### 3.8 Induced Collagen XII Knockdown Does Not Affect Cell Structure or Organization

Nuclear morphology and cell structure were assessed to determine if collagen XII is necessary for maintaining proper cell organization during postnatal development. There were no differences in cell density or nuclear orientation at either age (Figure S6A,B), and while the nuclear aspect ratio was marginally lower in p10 RosaCre‐KO tendons, distributions were similar at p30 (Figure S6C). F‐actin organization was also comparable between CTRL and RosaCre‐KO tendons at p10 and p30, with filaments arranged parallel to the longitudinal axis of the tendon (Figure S6D). Finally, in transverse sections, cell structure appeared normal in RosaCre‐KO tendons. Cell protrusions interacted with those of neighboring cells, forming clear boundaries for fibril bundles (Figure S6E). Altogether, these findings show that inducing collagen XII knockdown at birth did not affect cell organization or morphology during postnatal development, suggesting collagen XII is not required for maintaining cell shape and arrangement.

### 3.9 Induced Collagen XII Knockdown Has Minimal Effects on Collagen Fibril Morphology and Matrix Organization

**FIGURE 8:**
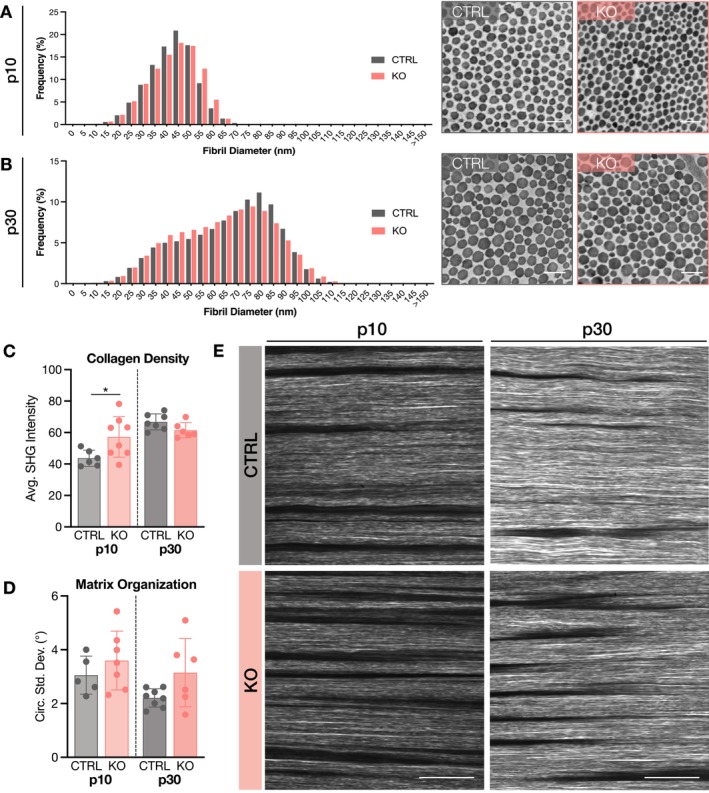
Induced collagen XII knockdown (RosaCre‐KO) has minimal effects on collagen fibril morphology and matrix organization. (A) The fibril diameter distribution of p10 RosaCre‐KO tendons shifted moderately towards larger fibril diameters compared to CTRL, while (B) the fibril diameter distribution of p30 RosaCre‐KO tendons was shifted towards smaller diameters compared to CTRL. Representative images of collagen fibrils shown on the right. Scale bar = 200 nm. (C) Collagen density was higher in p10 RosaCre‐KO tendons compared to CTRL, while (D) matrix disorganization was not different. (E) SHG images of the collagen matrix showed that organization in p10 and p30 RosaCre‐KO tendons was not qualitatively different from CTRL. Scale bar = 25 μm. Data presented as mean ± standard deviation (*p < 0.05).

Fibril morphology and matrix organization were evaluated to define the role of collagen XII in establishing tendon matrix structure. In p10 RosaCre‐KO tendons, collagen fibril diameters shifted towards larger fibril diameters (Figure 8A). In contrast, fibril diameters of p30 RosaCre‐KO tendons moderately shifted smaller compared to CTRL (Figure 8B), which may be attributed to lower body weight (Figure 7E). Additionally, at the fiber level, collagen density was higher in p10 RosaCre‐KO tendons with a small decrease in p30 KO tendons compared to CTRL (Figure 8C). Matrix organization was also comparable between genotypes in p30 tendons (Figure 8D,E).

### 3.10 Induced Collagen XII Knockdown Alters Mechanical Properties at the Tibial Insertion Site

**FIGURE 9:**
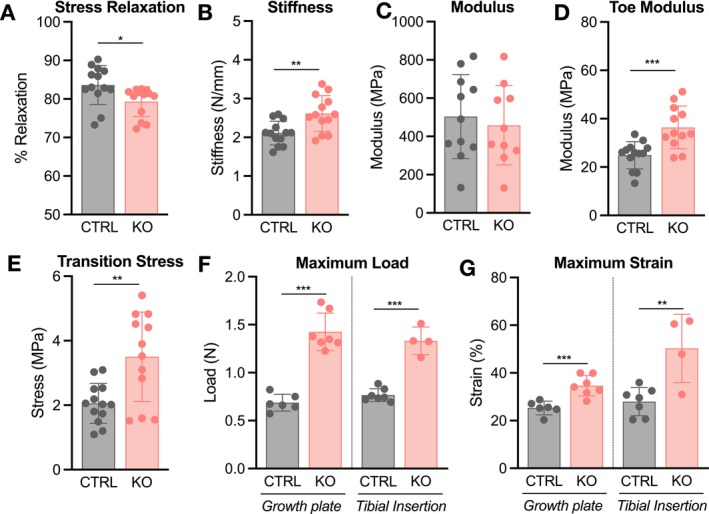
Induced collagen XII knockdown (RosaCre‐KO) increases tendon failure properties at p10. (A) RosaCre‐KO tendons had lower stress relaxation and (B) higher stiffness, (C) but there was no difference in midsubstance modulus. (D, E) Toe modulus and transition stress were higher in RosaCre‐KO tendons, along with (F) maximum load and (G) maximum strain at the growth plate and tibial insertion failure sites. Data presented as mean ± standard deviation (*p < 0.05, **p < 0.01, ***p < 0.001).

Despite minimal effects on matrix organization, induced collagen XII knockdown resulted in similar mechanical changes to ScxCre‐KO tendons. Cross‐sectional area and gauge length were not different in p10 RosaCre‐KO tendons (Figure S7). Similar to ScxCre‐KO tendons, percent relaxation was lower in RosaCre‐KO tendons (Figure 9A). And despite the higher stiffness in RosaCre‐KO tendons (Figure 9B), midsubstance modulus was not different when stain lines were optically tracked (Figure 9C). However, toe modulus and transition stress were higher in RosaCre‐KO tendons (Figure 9D,E). During the ramp to failure, failure modes in p10 tendons varied between the growth plate and tibial insertion; therefore, properties were stratified based on location. Maximum load and strain were higher in p10 RosaCre‐KO tendons, regardless of failure location (Figure 9F,G).

**FIGURE 10:**
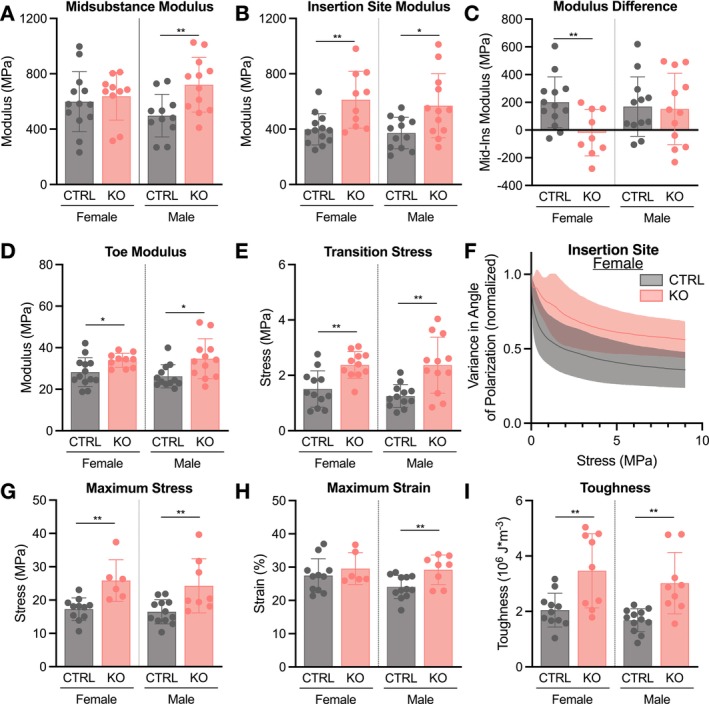
Induced collagen XII knockdown increases insertion site and failure mechanical properties at p30. (A) Midsubstance modulus was lower in male RosaCre‐KO tendons, while (B) insertion site modulus was higher in KO tendons of both sexes. (C) Modulus difference was only lower in female RosaCre‐KO tendons. (D) Toe modulus and (E) transition stress were higher in KO tendons of both sexes. (F) Collagen fiber realignment in the insertion site region of female mice demonstrating reduced alignment in the KO group. Solid lines are the mean with standard deviation represented by the shaded region. (G) Failure occurred at the tibial insertion site with increases in (G) maximum stress, (H) maximum strain, and (I) toughness in the RosaCre‐KO groups. Data presented as mean ± standard deviation (*p < 0.05, **p < 0.01, ***p < 0.001).

At p30, with the exception of smaller RosaCre‐KO tendons in males (Figure S8A), structural and viscoelastic properties, including gauge length, stiffness, and percent relaxation, were not different between CTRL and RosaCre‐KO tendons (Figure S8). While midsubstance modulus was only higher in male KO mice (Figure 10A), induced collagen XII knockdown led to similar mechanical changes observed in ScxCre‐KO mice, including increased insertion site modulus (Figure 10B), a diminished modulus difference in female RosaCre‐KO tendons (Figure 10C), increased toe modulus and transition stress (Figure 10D,E), reduced degree of collagen fiber realignment in the insertion site (Figures 10F and S9), and greater failure properties at the tibial insertion site (Figure 10G–I). Consistent with ScxCre‐KO mice, histological evaluation at the tibial insertion site demonstrated altered collagen fiber alignment at the enthesis with a reduction in mineralization (Figure S10). These findings point to a critical and distinct matrix assembly role of collagen XII following the establishment of cell organization.

### 3.11 Induced Collagen XII Knockdown Results in Minimal Gene Expression Changes

Despite the induction of significant *Col12a1* knockdown, gene expression changes were surprisingly minimal at both p10 and p30 (Figure S11A,B), suggesting no substantial changes in cell phenotype. Among the few changes, reduced *Gdf5* expression in p10 RosaCre‐KO tendons (Figure S11C) and increased *Mstn* expression in p30 RosaCre‐KO tendons were consistent with findings in ScxCre‐KO tendons (Figure S11D).

## 4 Discussion

The objective of this study was to define the temporal roles of collagen XII in regulating cell arrangement and matrix assembly in the establishment of tendon structure–function during development. Though classically defined as a modulator of fibrillogenesis and fibril surface properties, recent studies identified a novel cell‐mediated role of collagen XII [9, 24]. Therefore, we hypothesized that collagen XII regulates tendon cell arrangement during early development, prior to significant extracellular matrix deposition, which is pivotal for establishing normal structure–function. Overall, our results support our hypothesis that collagen XII plays a more substantial role in mediating cell arrangement than in the later stages of fibrillogenesis and subsequent matrix assembly. When collagen XII was knocked out throughout tendon development (ScxCre‐KO), patellar tendons were longer with altered F‐actin and matrix organization at the time of birth (p0), indicating a critical role prior to this developmental timepoint. Therefore, inducing knockdown of collagen XII at p0 (RosaCre‐KO) sought to isolate the specific developmental roles of collagen XII during matrix assembly. Changes in overall tendon and cell morphology of RosaCre‐KO mice were minimal, with few differences in tendon length, cell density, nuclear aspect ratio, F‐actin organization, and cell structure at p10 and p30. Mechanical differences at the tibial insertion site, however, were comparable between ScxCre‐KO and RosaCre‐KO mice, supporting a distinct matrix assembly role of collagen XII.

Increased length in ScxCre‐KO tendons was consistent across all ages. While tendon lengthening is often associated with a degenerative response due to a weakened matrix, such as following injury, longer patellar tendons at p0 suggest alterations during embryonic development prior to the deposition of significant matrix and the initiation of ambulation and mechanical loading. Sustained increased length at older ages could be related to impaired development of the tibial attachment, where the absence of an anchoring point disrupts the mechanical cues necessary for proper growth. Tuberosity formation requires contributions from both tendons and muscles [25, 26]. Muscle was not evaluated in the present study since we previously demonstrated that both mature tendon‐targeted scleraxis‐Cre and global collagen XII knockout mice have reduced grip strength, indicating muscle weakness [8, 10]. Reduced muscle loading could contribute to impaired formation of the tibial attachment, similar to compromised development of the deltoid tuberosity observed in mouse models of muscle weakness [25, 27, 28]. Alternatively, since we observed disruptions in tendon and tuberosity development as early as postnatal day 0 in ScxCre‐KO tendon and few changes in RosaCre‐KO tendons, a cell patterning mechanism intrinsic to the tendon could also be driving these phenotypic changes. Deltoid and teres major entheses demonstrated distinct expression domains in tendon and fibrocartilage of the developing enthesis, including *Col12a1*, *Tnc*, *Gli1*, and *Col1a1* [29], suggesting that the absence of *Col12a1* may have a direct effect on normal enthesis development.

Disruptions to cell organization and structure support previous work in mature global knockout mice [9], where collagen XII knockout inhibited the formation of cell protrusions. Though collagen fibers are present at p0, the striking alterations in cell structure at this age indicate a stronger cell‐mediated role of collagen XII. Cell protrusions in p0 and p10 ScxCre‐KO tendons were not truncated as seen in mature mice but were instead disorganized and failed to form interactions with neighboring cells. Our previous in vitro study demonstrated that collagen XII localizes to the processes that connect neighboring cells and forms bridges prior to cells establishing a physical filamentous actin connection [9]. Combined with results in the present study, this supports the critical role of collagen XII in establishing an effective tenocyte network in vivo. This tenocyte network is critical for coordinating a cell's ability to sense and respond to mechanical loads, which is regulated by both cell–cell and cell–matrix interactions [30, 31]. The observation of disrupted connections between neighboring cells in ScxCre‐KO p0 tendons indicates abnormalities in cell–cell interactions. However, knockdown of collagen XII at p0 did not induce the same alterations in F‐actin organization or cell structure compared to ScxCre‐KO tendons, suggesting that collagen XII is necessary for establishing initial cell–cell interactions but not required for maintaining them. Furthermore, increased tendon length and the disruption of F‐actin organization and nuclear morphology in ScxCre‐KO tendons suggest cells may be de‐tensioned in their matrix environment, leading to an altered nuclear and cell phenotype, as demonstrated by gene expression changes in tendon and cartilage markers.

Additionally, the number of fibripositors decreases during postnatal development [32], but their abundance in p10 ScxCre‐KO tendons indicates an aberrant fibrillogenesis process, which impairs both linear and lateral growth processes and results in slightly smaller fibril diameters by postnatal Day 30. In RosaCre‐KO mice, fibril diameter distributions were moderately different at both ages, with a shift towards larger fibril diameters in p10 tendons and smaller fibril diameters in p30 tendons. However, p30 RosaCre‐KO mice were smaller, which may account for the observation of smaller fibrils. In contrast to other assays, fibril morphology results may point to the specific, matrix assembly role in regulating fibril lateral growth. RosaCre‐KO tendons in p10 mice demonstrated a similar but less substantial shift towards larger fibril diameters compared to p10 ScxCre‐KO tendons, and this shift is consistent with later timepoints in the global knockout model [9] as well as in other tissues, such as skin [33]. Given its fibril‐associated nature, these results support that collagen XII plays a role in determining fibril diameter.

In mature ScxCre‐KO mice, mechanical properties in FDL and patellar tendons are impaired due to the absence of collagen XII, with reduced stiffness, dynamic modulus, and degree of collagen fiber realignment with loading [10]. We therefore hypothesized that mechanical function would also be disrupted at younger ages due to a disorganized matrix. Surprisingly, both ScxCre‐ and RosaCre‐KO p10 tendons demonstrated properties characteristic of increased elasticity, including an approximately 30% higher modulus and stiffness in ScxCre‐ and RosaCre‐KO mice, respectively. Both groups also exhibited reduced percent relaxation and increases in failure properties. While quantitative polarized light imaging analysis could not be conducted at p10, the consistent increases in toe modulus and transition stress across both ages in ScxCre‐ and RosaCre‐KO tendons suggest corresponding changes in fiber loading mechanisms that may explain higher stiffness and modulus. Transverse sections showed aberrant fiber structure when cell protrusions fail to connect with neighboring cells, and this may affect the ability for fibers to uncrimp, slide, and deform throughout the toe and linear regions. CTRL and ScxCre‐KO tendons had similar matrix alignment by p30, but areas of disorganized matrix suggest that imaging at this scale is not sensitive to detect higher‐order differences in matrix organization that were later observed via collagen fiber realignment in mature day 60 mice. In clinical presentations of myopathic EDS, some symptoms, such as muscle weakness, appear to resolve during early adulthood [34, 35], further supporting a more prominent cell‐mediated mechanism during early development. Future studies are required to further explore the potential impact of collagen XII on collagen fiber loading mechanisms.

Most mechanical changes occurred in the insertion site region, with increases in modulus and failure properties at both p10 and p30 as well as altered collagen fiber realignment at p30. These changes could be attributed to impairment of the tibial attachment that was observed histologically. As the tendon enthesis consists of a gradient from bone to tendon, compositional differences result in a lower modulus closer to the insertion site, and the gradient between the insertion site and tendon midsubstance minimizes stress concentrations. In the absence of collagen XII, the lack of a true tendon enthesis may have led to the diminished modulus difference between the insertion site and midsubstance. Additionally, because we observed rounder nuclei and impaired spindle‐like structure of cells in ScxCre‐KO tendons, we were surprised that the expression of genes associated with cartilage and fibrocartilage, such as *Gdf5*, *Col2a1*, *Sox9*, and *Acan* was lower. Instead, these findings could be explained by disruptions to the development of the insertion site that may affect the composition of this region [36]. Though a scleraxis‐Cre driver is intended to specifically target knockout to tendons, a limitation of this mouse model is that studies have shown ScxCre expression in the patella, growth plate cartilage, muscle, bone, and the periosteum [37, 38]. Therefore, collagen XII knockout in these regions, particularly near the tendon insertion site, could be driving these differences rather than isolated knockout to tendon cells. Limitations of using a global inducible model are similar, and reduced body weight in RosaCre‐KO mice suggests systemic effects that do not solely target tendons, consistent with the global collagen XII knockout mouse model [24]. Nevertheless, pilot work and previous studies have shown that an inducible scleraxis‐Cre model is not effective for gene excision, and the ubiquitous Rosa model remains the most effective model for investigating the temporal roles of matrix proteins using genetic knockdown [39, 40].

Finally, increased tendon length, as well as greater fibril diameter and cell density in p10 and p30 ScxCre‐KO tendons, respectively, point to a possible hypertrophic phenotype due to collagen XII knockout. Cornea and skin studies showed that because of its localization to the fibril surface, collagen XII may be necessary for storing latent TGF‐β [33, 41]. In its absence, there is reduced latent storage and increased TGF‐β availability and activity in corneas, resulting in a hypertrophic response of increased collagen density as well as expression of the TGF‐β responsive genes, *Serpine1*, *Col1a1*, and *Col5a1*. Gene expression findings in this study support a similar mechanism in tendon, where TGF‐β responsive genes such as *Mstn* and *Serpine1* are also upregulated despite no changes in *Tgfb1, 2*, or *3* expression. It is possible that short and long collagen XII isoforms may play different roles in interactions with the extracellular matrix [42]. For example, the long isoform may have a greater structural role in modulating fibril and fiber organization, while the short isoform could be responsible for interacting with other molecules, such as TGF‐β. The conditional *Col12a1* ^flox/flox^ mouse used in this study reduces expression of both isoforms [10], and additional studies are necessary to understand the differential roles of both isoforms and determine whether active TGF‐β is indeed increased due to the absence of collagen XII. Interestingly, we did not identify a compensatory upregulation in the gene expression of the related FACIT, or *Col14a1*, in response to collagen XII knockout. Instead, expression of many collagenous gene decreased, including *Col14a1* and *Col2a1* at p10 and *Col1a1* and *Col1a2* at p30. Knockout of collagen XII primarily results in the upregulation of genes encoding regulatory and signaling glycoproteins, such as *Mstn*, *Thbs4, Cd44*, and *Postn*, further supporting a predominant role in modulating cell‐mediated processes rather than a structural matrix component.

In summary, our results demonstrate a critical role of collagen XII in regulating cell arrangement during tendon development. In tendon‐targeted scleraxis‐Cre collagen XII knockout mice, we found profound changes in cell organization and structure at postnatal day 0 as well as alterations in matrix organization and structure at later postnatal time points. However, when collagen XII knockdown was induced at postnatal day 0, the effects on matrix organization and structure were minimal. Our findings have important implications for understanding the coordinated cell–cell and cell–matrix mechanisms that are necessary to re‐create the native tendon structure following tendon injury.

## Author Contributions

A.K. Fung, N.A. Dyment, and L.J. Soslowsky designed the study. A.K. Fung, S.N. Weiss, C.A. Nuss, and W. Yen collected and analyzed the data. A.K. Fung, S.W. Volk, N.A. Dyment, and L.J. Soslowsky interpreted the findings. A.K. Fung drafted the manuscript. A.K. Fung, S.N. Weiss, C.A. Nuss, W. Yen, S.W. Volk, N.A. Dyment, and L.J. Soslowsky reviewed and edited the manuscript. All authors read and approved the final version.

## Conflicts of Interest

The authors declare no conflicts of interest.

## Supporting information


**Figure S1:** When p30 tendons were stratified by sex, the rounded nuclei observed in ScxCre‐KO tendons was largely influenced by the results in males. TEM images of (B) p0 CTRL tendon and (C,D) p0 ScxCre‐KO tendons. (D′) Fibripositors are indicated by red arrows. TEM images of (E) p10 CTRL tendon and (F,G) p10 ScxCre‐KO tendons. (G') Fibripositors are indicated by red arrows. Scale bar = 1 μm.

**Figure S2:** (A) Cross‐sectional area, (B) gauge length, (C) stiffness, and (D) maximum strain of p10 CTRL and ScxCre‐KO tendons. Data presented as mean ± standard deviation (***p < 0.001).

**Figure S3:** (A) Dynamic modulus was not different at any age, while (B) phase shift was lower in p10 and female p30 ScxCre‐KO tendons. Data presented as mean ± standard deviation (*p < 0.05).

**Figure S4:** (A) Cross‐sectional area, (B) gauge length, (C) stiffness, and (D) percent relaxation in p30 ScxCre‐KO tendons. Data presented as mean ± standard deviation (*p < 0.05, ***p < 0.001). (E,F) In the midsubstance, collagen fiber realignment was not different. Solid lines are the mean with standard deviation represented by the shaded region.

**Figure S5:** (A) SHG of CTRL tendons with a clear tidemark noted by the red arrow. (B) Mineral deposition (calcein blue) and mineralizing cells (alkaline phosphatase, AP, yellow) in CTRL tendons. (C) SHG of ScxCre‐KO tendons showing lack of a clear tidemark, and (D) mineral deposition is impaired in KO tendons. Scale bar = 100 μm.

**Figure S6:** There were no differences in (A) cell density, (B) nuclear orientation, or (C) nuclear aspect ratio between CTRL and RosaCre‐KO tendons at either age. (D) F‐actin organization in p10 and p30 RosaCre‐KO mice appeared normal, with F‐actin arranged parallel with the longitudinal axis of the tendon. Scale bar = 25 μm. (E) Cell and fiber structure were not affected in RosaCre‐KO tendons at either age. Cell protrusions interacted with neighboring cells forming boundaries around fibril bundles. Scale bar = 1 μm.

**Figure S7:** (A) Cross‐sectional area and (B) gauge length of p10 RosaCre‐KO tendons. Data presented as mean ± standard deviation.

**Figure S8:** (A) Cross‐sectional area, (B) gauge length, (C) stiffness, and (D) percent relaxation in p30 CTRL and RosaKO tendons (E) Dynamic modulus was higher in p10 RosaKO tendons, but (F) there were no differences in phase shift at either age. Data presented as mean ± standard deviation (*p < 0.05).

**Figure S9:** (A) In the insertion site of male mice, RosaCre‐KO tendons demonstrated less realignment, but collagen fiber realignment was not different in the midsubstance of either (B) female or (C) male RosaCre‐KO tendons. Solid lines are the mean with standard deviation represented by the shaded region.

**Figure S10:** (A) SHG of CTRL tendons (B) Mineral deposition (calcein blue) and mineralizing cells (alkaline phosphatase, AP, yellow) in CTRL tendons. (C) SHG of RosaCre‐KO tendons showing disrupted collagen fiber organization, and (D) mineral deposition is impaired in KO tendons. Scale bar = 100 μm.

**Figure S11:** (A,B) Principal component analysis did not demonstrate clustering based on genotype at p10 and p30. (C,D) Volcano plots showed minimal changes in gene expression at p10 and p30. At p10, RosaCre‐KO tendons had lower expression of *Gdf5* and *Loxl2*, while at p30, RosaCre‐KO tendons had lower expression of *Acan* and *Fn1* and higher expression of *Mstn*.

**Table S1:** TaqMan assays for gene expression.

**Table S2:** Gene expression raw data.

## Data Availability

The authors have nothing to report.
