# Supplementary material for: Collagen XII Plays a More Prominent Cell‐Mediated Role in Tendon Organization Compared to Matrix Assembly During Postnatal Development
Source: FASEB J. 2025 Oct 29;39(21):e71196. doi: 10.1096/fj.202501618R (PMC12571144; doi:10.1096/fj.202501618R)
Supplement: Supplementary file 1 — Figure S1: When p30 tendons were stratified by sex, the rounded nuclei observed in ScxCre‐KO tendons was largely influenced by the results in males. TEM images of (B) p0 CTRL tendon and (C,D) p0 ScxCre‐KO tendons. (D′) Fibripositors are indicated by red arrows. TEM images of (E) p10 CTRL tendon and (F,G) p10 ScxCre‐KO tendons. (G') Fibripositors are indicated by red arrows. Scale bar = 1 μm. [file FSB2-39-e71196-s009.pdf]

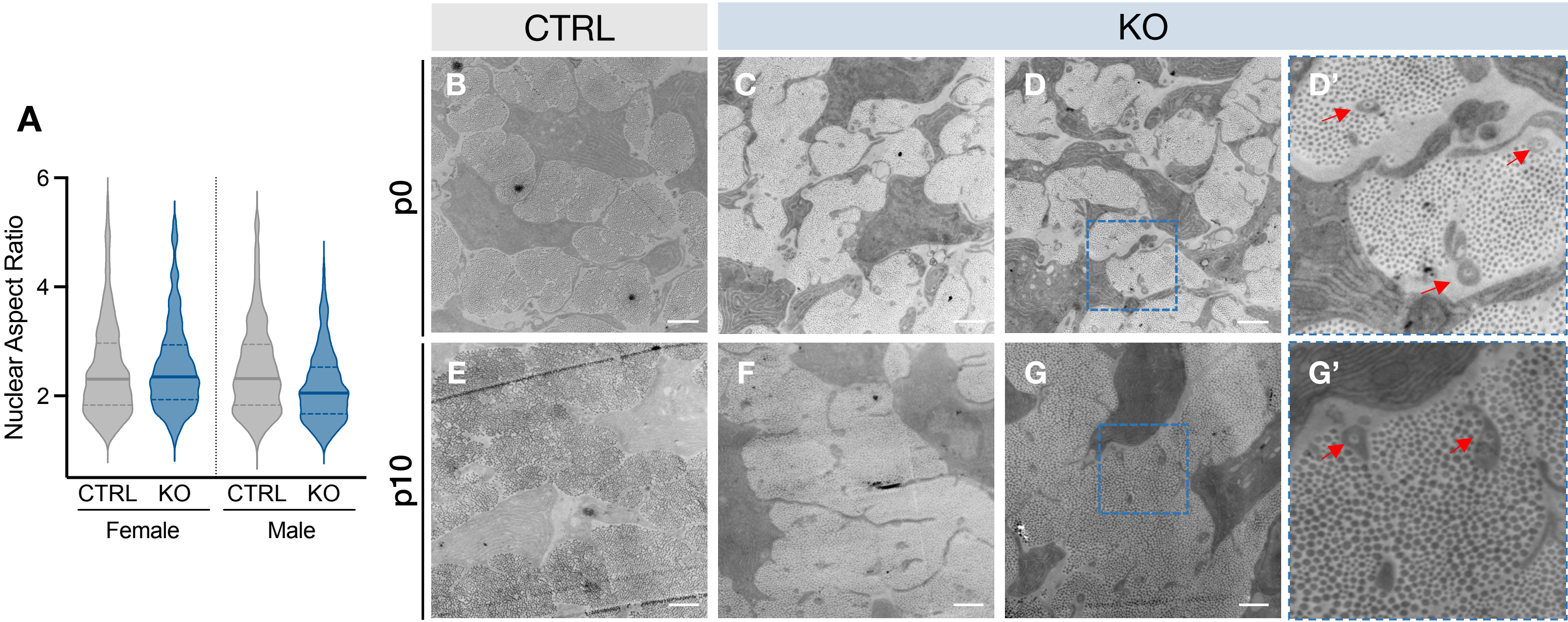

Supplemental Figure 1. When p30 tendons were stratified by sex, the rounded nuclei observed in ScxCre-KO tendons was largely influenced by the results in males. TEM images of B) p0 CTRL tendon and C,D) p0 ScxCre-KO tendons. D') Fibripositors are indicated by red arrows. TEM images of E) p10 CTRL tendon and F,G) p10 ScxCre-KO tendons. G') Fibripositors are indicated by red arrows. Scale bar = 1µm.
