# Supplementary material for: Collagen XII Plays a More Prominent Cell‐Mediated Role in Tendon Organization Compared to Matrix Assembly During Postnatal Development
Source: FASEB J. 2025 Oct 29;39(21):e71196. doi: 10.1096/fj.202501618R (PMC12571144; doi:10.1096/fj.202501618R)
Supplement: Supplementary file 2 — Figure S2: (A) Cross‐sectional area, (B) gauge length, (C) stiffness, and (D) maximum strain of p10 CTRL and ScxCre‐KO tendons. Data presented as mean ± standard deviation (***p < 0.001). [file FSB2-39-e71196-s008.pdf]

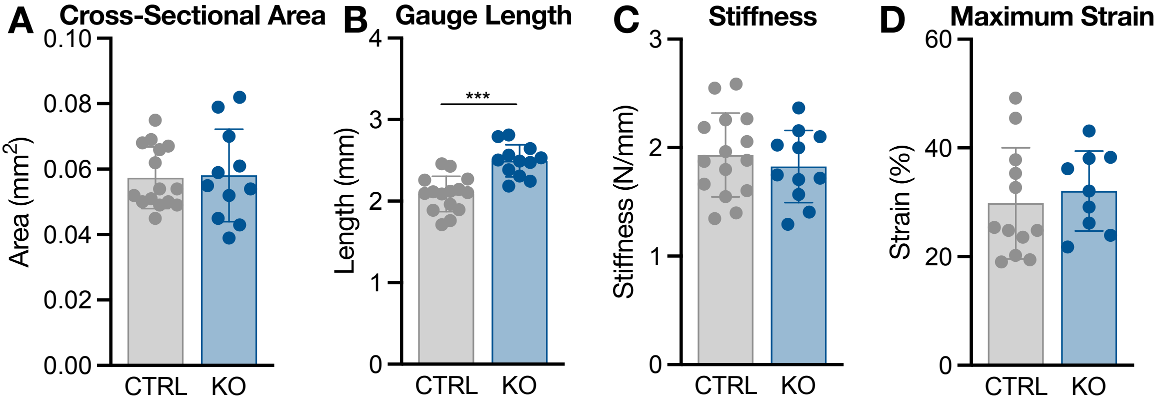

Supplemental Figure 2. A) Cross-sectional area, B) gauge length, C) stiffness, and D) maximum strain of p10 CTRL and ScxCre-KO tendons. Data presented as mean ± standard deviation (***$p<0.001$).
