# Supplementary material for: Collagen XII Plays a More Prominent Cell‐Mediated Role in Tendon Organization Compared to Matrix Assembly During Postnatal Development
Source: FASEB J. 2025 Oct 29;39(21):e71196. doi: 10.1096/fj.202501618R (PMC12571144; doi:10.1096/fj.202501618R)
Supplement: Supplementary file 3 — Figure S3: (A) Dynamic modulus was not different at any age, while (B) phase shift was lower in p10 and female p30 ScxCre‐KO tendons. Data presented as mean ± standard deviation (*p < 0.05). [file FSB2-39-e71196-s002.pdf]

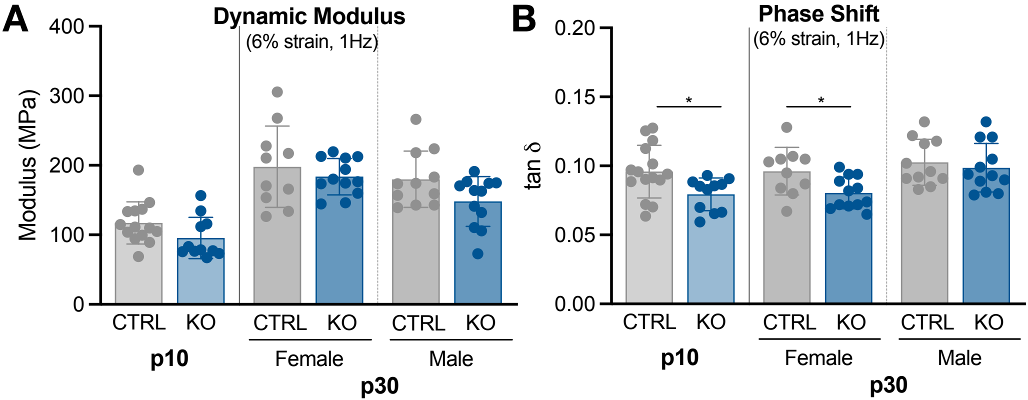

Supplemental Figure 3. A) Dynamic modulus was not different at any age, while B) phase shift was lower in p10 and female p30 ScxCre-KO tendons. Data presented as mean ± standard deviation (*$p<0.05$).
