# Supplementary material for: Collagen XII Plays a More Prominent Cell‐Mediated Role in Tendon Organization Compared to Matrix Assembly During Postnatal Development
Source: FASEB J. 2025 Oct 29;39(21):e71196. doi: 10.1096/fj.202501618R (PMC12571144; doi:10.1096/fj.202501618R)
Supplement: Supplementary file 4 — Figure S4: (A) Cross‐sectional area, (B) gauge length, (C) stiffness, and (D) percent relaxation in p30 ScxCre‐KO tendons. Data presented as mean ± standard deviation (*p < 0.05, ***p < 0.001). (E,F) In the midsubstance, collagen fiber realignment was not different. Solid lines are the mean with standard deviation represented by the shaded region. [file FSB2-39-e71196-s006.pdf]

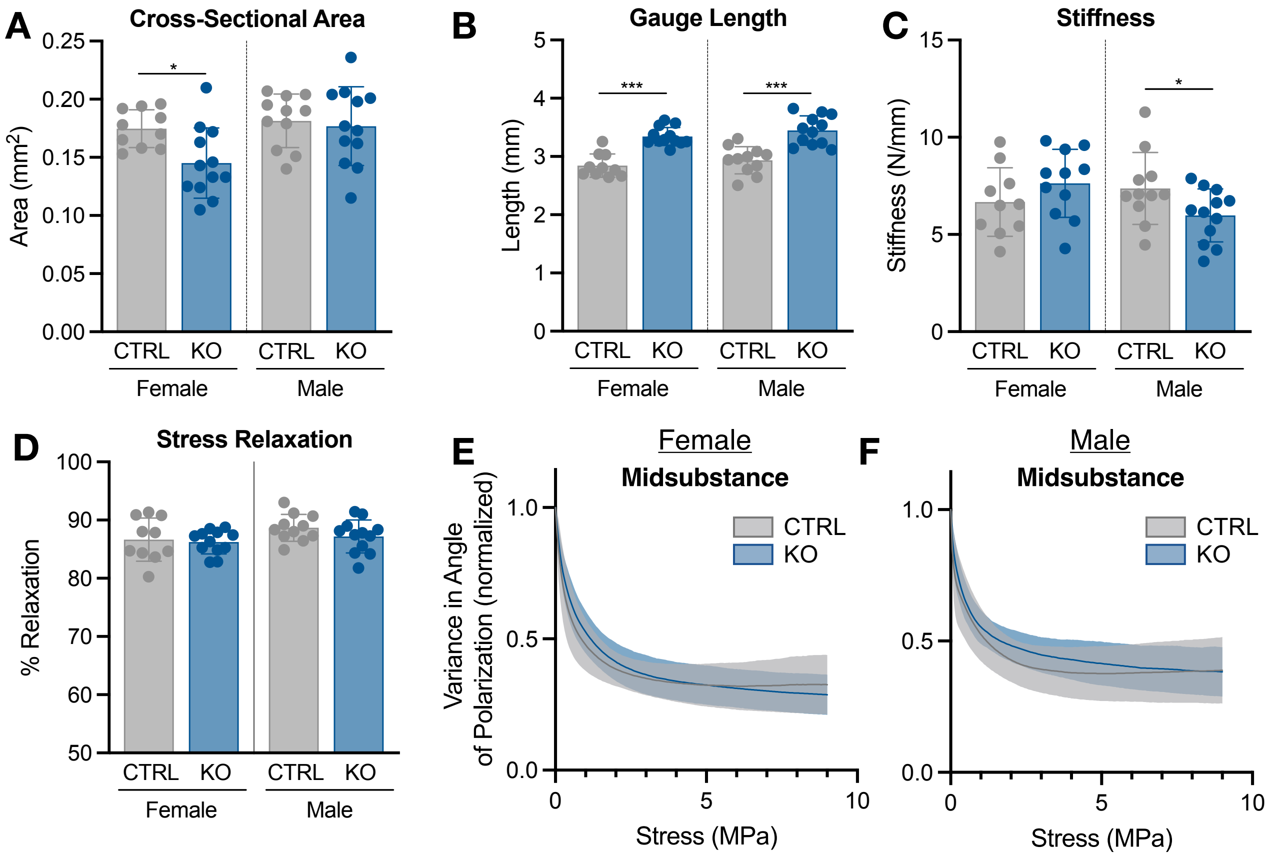

Supplemental Figure 4. A) Cross-sectional area, B) gauge length, C) stiffness, and D) percent relaxation in p30 ScxCre-KO tendons. Data presented as mean ± standard deviation (*p<0.05, ***p<0.001). E-F) In the midsubstance, collagen fiber realignment was not different. Solid lines are the mean with standard deviation represented by the shaded region.
