# Supplementary material for: Collagen XII Plays a More Prominent Cell‐Mediated Role in Tendon Organization Compared to Matrix Assembly During Postnatal Development
Source: FASEB J. 2025 Oct 29;39(21):e71196. doi: 10.1096/fj.202501618R (PMC12571144; doi:10.1096/fj.202501618R)
Supplement: Supplementary file 5 — Figure S5: (A) SHG of CTRL tendons with a clear tidemark noted by the red arrow. (B) Mineral deposition (calcein blue) and mineralizing cells (alkaline phosphatase, AP, yellow) in CTRL tendons. (C) SHG of ScxCre‐KO tendons showing lack of a clear tidemark, and (D) mineral deposition is impaired in KO tendons. Scale bar = 100 μm. [file FSB2-39-e71196-s005.pdf]

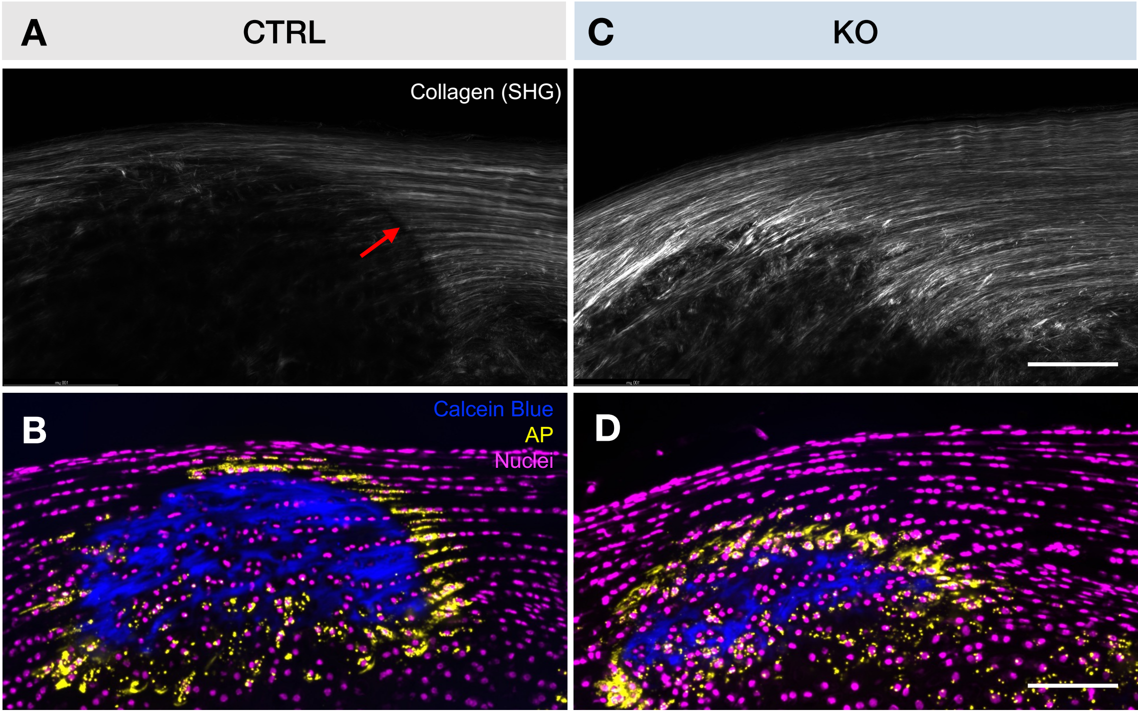

Supplemental Figure 5. A) SHG of CTRL tendons with a clear tidemark noted by the red arrow. B) Mineral deposition (calcein blue) and mineralizing cells (alkaline phosphatase, AP, yellow) in CTRL tendons. C) SHG of ScxCre-KO tendons showing lack of a clear tidemark, and D) mineral deposition is impaired in KO tendons. Scale bar = 100μm.
