# Supplementary material for: Collagen XII Plays a More Prominent Cell‐Mediated Role in Tendon Organization Compared to Matrix Assembly During Postnatal Development
Source: FASEB J. 2025 Oct 29;39(21):e71196. doi: 10.1096/fj.202501618R (PMC12571144; doi:10.1096/fj.202501618R)
Supplement: Supplementary file 6 — Figure S6: There were no differences in (A) cell density, (B) nuclear orientation, or (C) nuclear aspect ratio between CTRL and RosaCre‐KO tendons at either age. (D) F‐actin organization in p10 and p30 RosaCre‐KO mice appeared normal, with F‐actin arranged parallel with the longitudinal axis of the tendon. Scale bar = 25 μm. (E) Cell and fiber structure were not affected in RosaCre‐KO tendons at either age. Cell protrusions interacted with neighboring cells forming boundaries around fibril bundles. Scale bar = 1 μm. [file FSB2-39-e71196-s012.pdf]

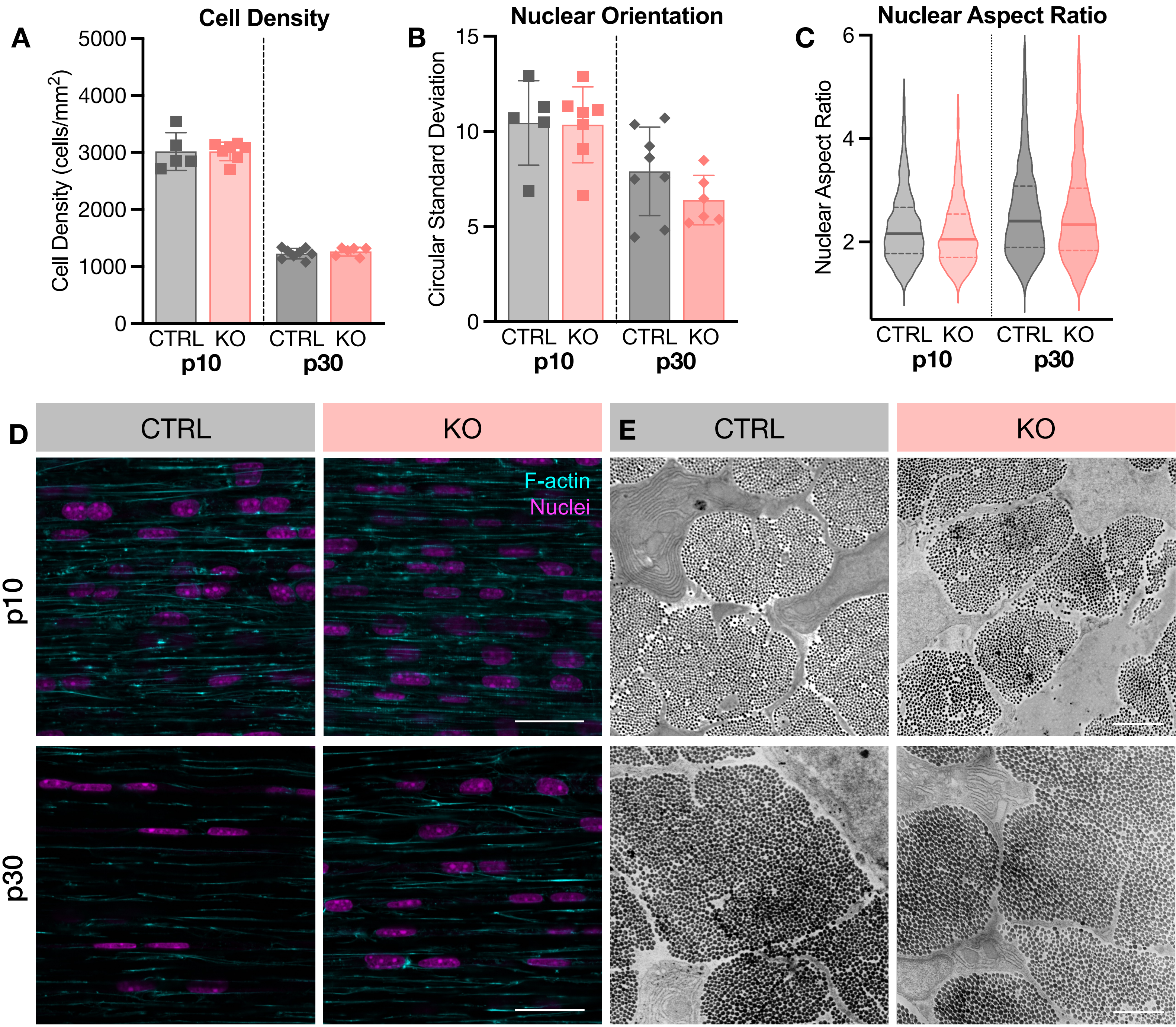

Supplemental Figure 6. There were no differences in A) cell density, B) nuclear orientation, or C) nuclear aspect ratio between CTRL and RosaCre-KO tendons at either age. D) F-actin organization in p10 and p30 RosaCre-KO mice appeared normal, with F-actin arranged parallel with the longitudinal axis of the tendon. Scale bar = 25μm. E) Cell and fiber structure were not affected in RosaCre-KO tendons at either age. Cell protrusions interacted with neighboring cells forming boundaries around fibril bundles. Scale bar = 1μm.
