# Supplementary material for: Collagen XII Plays a More Prominent Cell‐Mediated Role in Tendon Organization Compared to Matrix Assembly During Postnatal Development
Source: FASEB J. 2025 Oct 29;39(21):e71196. doi: 10.1096/fj.202501618R (PMC12571144; doi:10.1096/fj.202501618R)
Supplement: Supplementary file 7 — Figure S7: (A) Cross‐sectional area and (B) gauge length of p10 RosaCre‐KO tendons. Data presented as mean ± standard deviation. [file FSB2-39-e71196-s003.pdf]

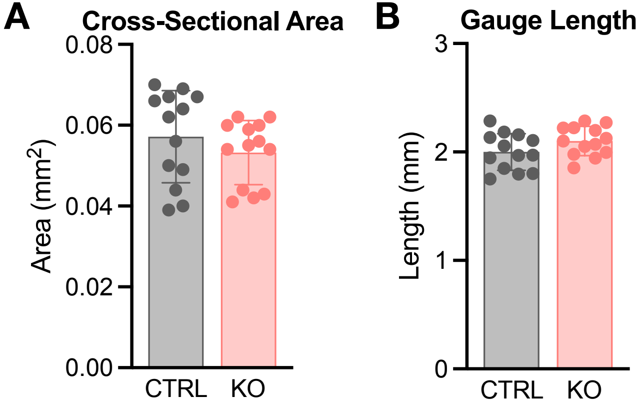

Supplemental Figure 7. A) Cross-sectional area and B) gauge length of p10 RosaCre-KO tendons. Data presented as mean ± standard deviation.
