# Supplementary material for: Collagen XII Plays a More Prominent Cell‐Mediated Role in Tendon Organization Compared to Matrix Assembly During Postnatal Development
Source: FASEB J. 2025 Oct 29;39(21):e71196. doi: 10.1096/fj.202501618R (PMC12571144; doi:10.1096/fj.202501618R)
Supplement: Supplementary file 8 — Figure S8: (A) Cross‐sectional area, (B) gauge length, (C) stiffness, and (D) percent relaxation in p30 CTRL and RosaKO tendons (E) Dynamic modulus was higher in p10 RosaKO tendons, but (F) there were no differences in phase shift at either age. Data presented as mean ± standard deviation (*p < 0.05). [file FSB2-39-e71196-s001.pdf]

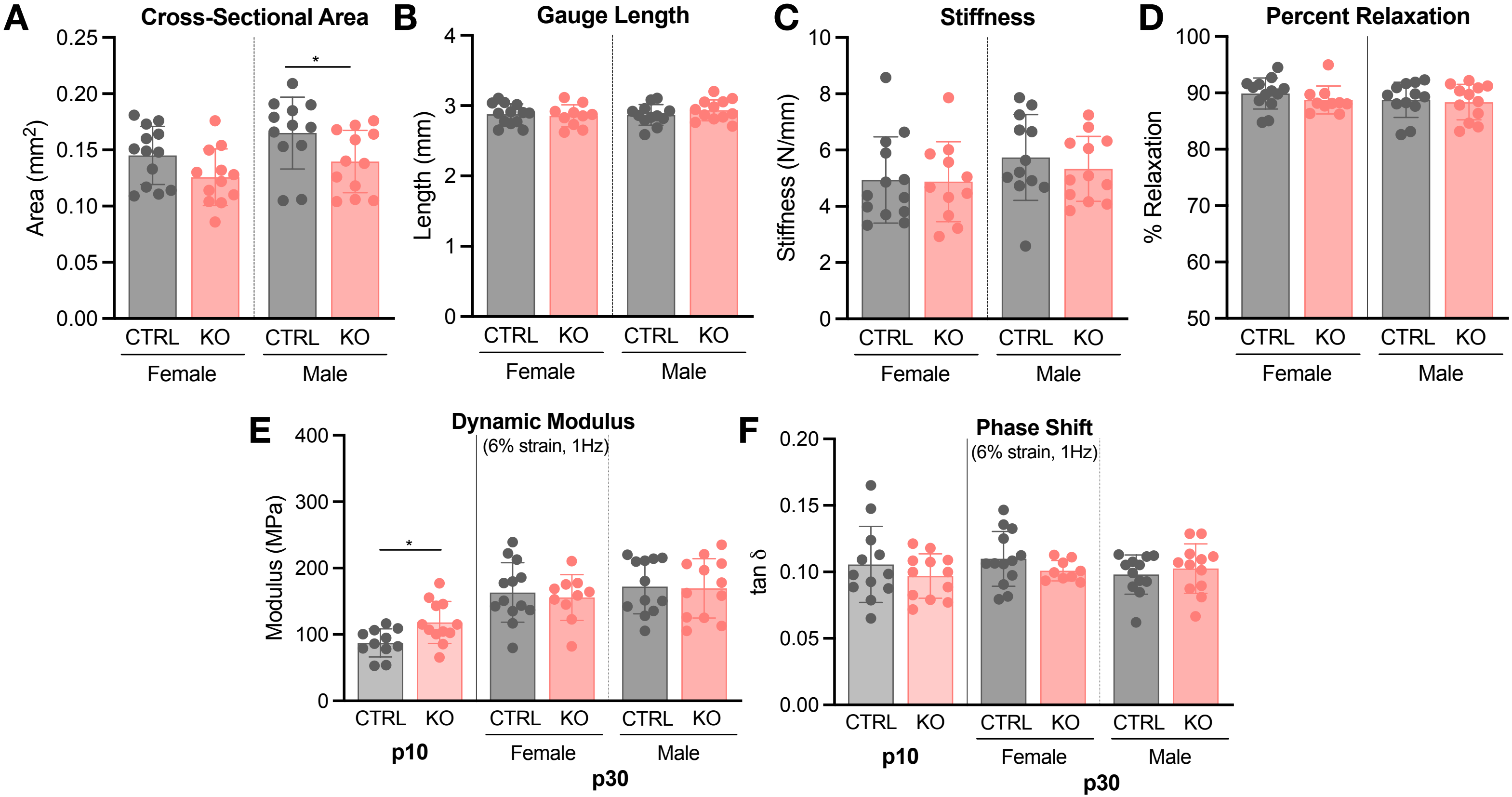

Supplemental Figure 8. A) Cross-sectional area, B) gauge length, C) stiffness, and D) percent relaxation in p30 CTRL and RosaKO tendons E) Dynamic modulus was higher in p10 RosaKO tendons, but F) there were no differences in phase shift at either age. Data presented as mean ± standard deviation (*$p<0.05$).
