# Supplementary material for: Collagen XII Plays a More Prominent Cell‐Mediated Role in Tendon Organization Compared to Matrix Assembly During Postnatal Development
Source: FASEB J. 2025 Oct 29;39(21):e71196. doi: 10.1096/fj.202501618R (PMC12571144; doi:10.1096/fj.202501618R)
Supplement: Supplementary file 9 — Figure S9: (A) In the insertion site of male mice, RosaCre‐KO tendons demonstrated less realignment, but collagen fiber realignment was not different in the midsubstance of either (B) female or (C) male RosaCre‐KO tendons. Solid lines are the mean with standard deviation represented by the shaded region. [file FSB2-39-e71196-s010.pdf]

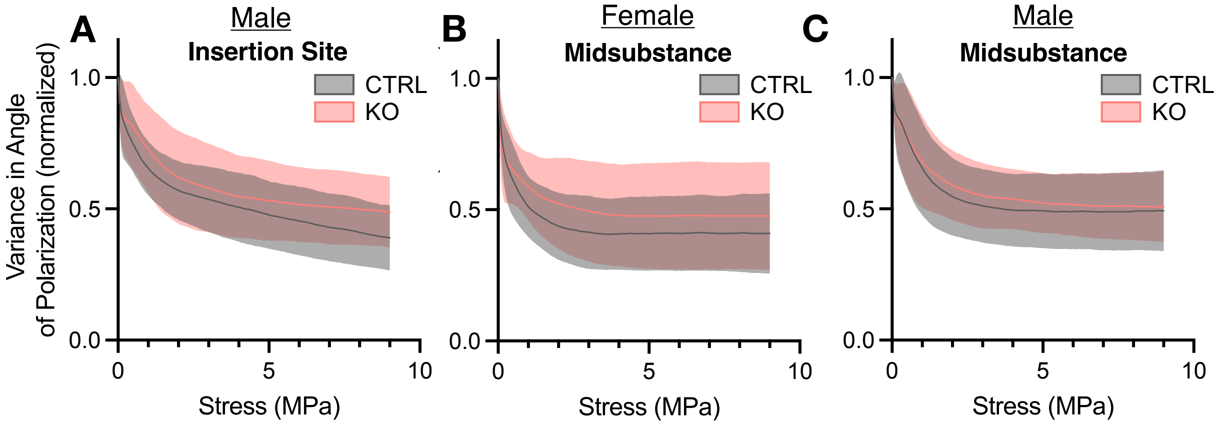

Supplemental Figure 9. A) In the insertion site of male mice, RosaCre-KO tendons demonstrated less realignment, but collagen fiber realignment was not different in the midsubstance of either B) female or C) male RosaCre-KO tendons. Solid lines are the mean with standard deviation represented by the shaded region.
