# Supplementary material for: Collagen XII Plays a More Prominent Cell‐Mediated Role in Tendon Organization Compared to Matrix Assembly During Postnatal Development
Source: FASEB J. 2025 Oct 29;39(21):e71196. doi: 10.1096/fj.202501618R (PMC12571144; doi:10.1096/fj.202501618R)
Supplement: Supplementary file 10 — Figure S10: (A) SHG of CTRL tendons (B) Mineral deposition (calcein blue) and mineralizing cells (alkaline phosphatase, AP, yellow) in CTRL tendons. (C) SHG of RosaCre‐KO tendons showing disrupted collagen fiber organization, and (D) mineral deposition is impaired in KO tendons. Scale bar = 100 μm. [file FSB2-39-e71196-s013.pdf]

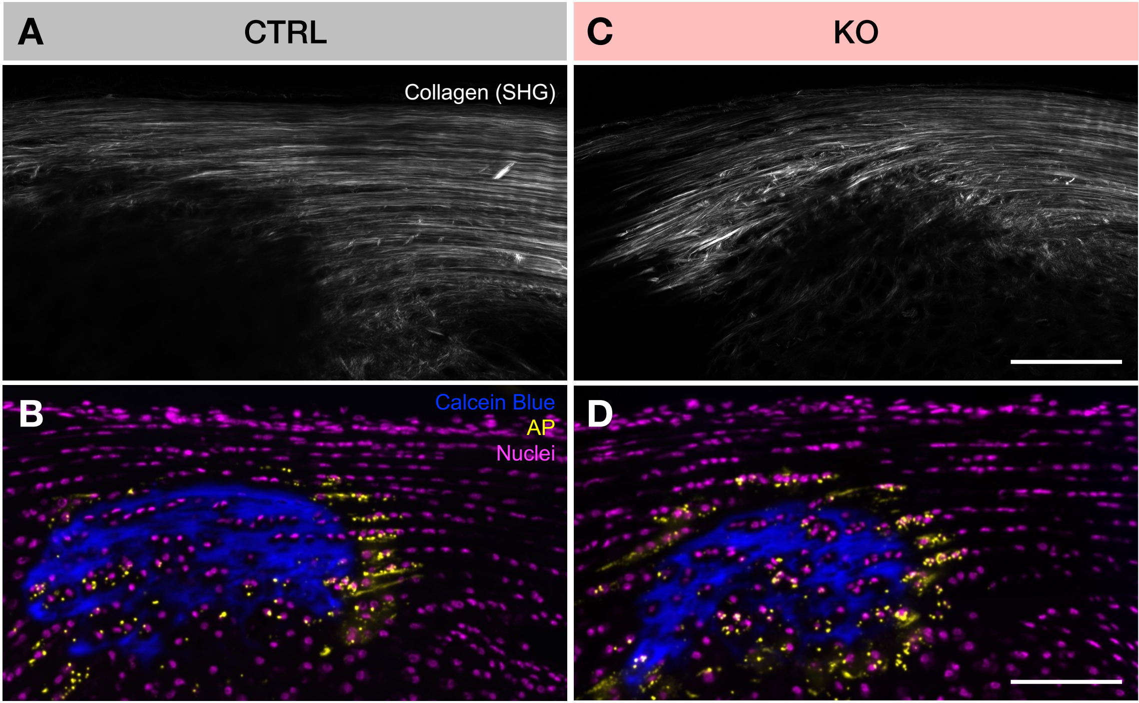

Supplemental Figure 10. A) SHG of CTRL tendons B) Mineral deposition (calcein blue) and mineralizing cells (alkaline phosphatase, AP, yellow) in CTRL tendons. C) SHG of RosaCre-KO tendons showing disrupted collagen fiber organization, and D) mineral deposition is impaired in KO tendons. Scale bar = 100μm.
