# Supplementary material for: Collagen XII Plays a More Prominent Cell‐Mediated Role in Tendon Organization Compared to Matrix Assembly During Postnatal Development
Source: FASEB J. 2025 Oct 29;39(21):e71196. doi: 10.1096/fj.202501618R (PMC12571144; doi:10.1096/fj.202501618R)
Supplement: Supplementary file 11 — Figure S11: (A,B) Principal component analysis did not demonstrate clustering based on genotype at p10 and p30. (C,D) Volcano plots showed minimal changes in gene expression at p10 and p30. At p10, RosaCre‐KO tendons had lower expression of Gdf5 and Loxl2, while at p30, RosaCre‐KO tendons had lower expression of Acan and Fn1 and higher expression of Mstn. [file FSB2-39-e71196-s011.pdf]

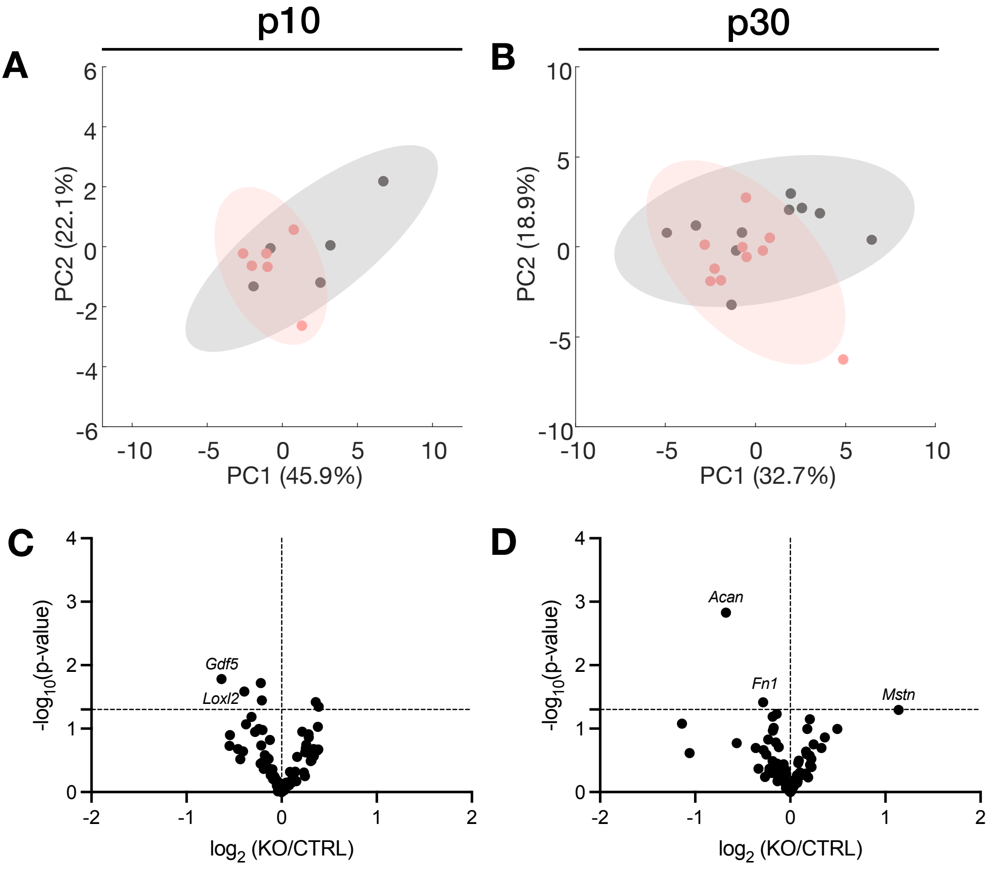

Supplemental Figure 11. A-B) Principal component analysis did not demonstrate clustering based on genotype at p10 and p30. C-D) Volcano plots showed minimal changes in gene expression at p10 and p30. At p10, RosaCre-KO tendons had lower expression of *Gdf5* and *Loxl2*, while at p30, RosaCre-KO tendons had lower expression of *Acan* and *Fn1* and higher expression of *Mstn*.
