# Supplementary material for: Collagen XII Plays a More Prominent Cell‐Mediated Role in Tendon Organization Compared to Matrix Assembly During Postnatal Development
Source: FASEB J. 2025 Oct 29;39(21):e71196. doi: 10.1096/fj.202501618R (PMC12571144; doi:10.1096/fj.202501618R)
Supplement: Supplementary file 12 — Table S1: TaqMan assays for gene expression. [file FSB2-39-e71196-s007.docx]

Supplemental Table 1: TaqMan Assays for Gene Expression

| **Category** | **Gene** | **TaqMan Assay ID** |  | **Category** | **Gene** | **TaqMan Assay ID** |
| --- | --- | --- | --- | --- | --- | --- |
| **Collagens** | *Col1a1* | Mm00801666_g1 |  | **Matrix Remodeling** | *Adamts1* | Mm01344169_m1 |
|  | *Col1a2* | Mm00483888_m1 |  |  | *Adamts2* | Mm00805170_m1 |
|  | *Col2a1* | Mm01309565_m1 |  |  | *Adamts5* | Mm00478620_m1 |
|  | *Col3a1* | Mm00802300_m1 |  |  | *Ext2* | Mm00468775_m1 |
|  | *Col4a1* | Mm01210125_m1 |  |  | *Lox* | Mm00495386_m1 |
|  | *Col5a1* | Mm00489289_m1 |  |  | *Loxl1* | Mm01145738_m1 |
|  | *Col5a2* | Mm00483675_m1 |  |  | *Loxl2* | Mm00804740_m1 |
|  | *Col6a1* | Mm00487160_m1 |  |  | *Mmp2* | Mm00439498_m1 |
|  | *Col10a1* | Mm00487041_m1 |  |  | *Mmp13* | Mm00439491_m1 |
|  | *Col11a1* | Mm00483387_m1 |  |  | *Mmp14* | Mm00485054_m1 |
|  | *Col12a1* | Mm01148556_m1 |  |  | *Mmp16* | Mm01210646_m1 |
|  | *Col14a1* | Mm00805269_m1 |  |  | *Timp1* | Mm01341361_m1 |
| **Proteoglycans** | *Acan* | Mm00545794_m1 |  |  | *Timp3* | Mm00441826_m1 |
|  | *Aspn* | Mm00445946_m1 |  | **Cell-cell & Cell-ECM Interactions** | *Cdh2* | Mm01162497_m1 |
|  | *Bgn* | Mm01191753_m1 |  |  | *Cdh11* | Mm00515466_m1 |
|  | *Cspg4* | Mm00507257_m1 |  |  | *Gja1* | Mm00439105_m1 |
|  | *Dcn* | Mm00514535_m1 |  |  | *Itga5* | Mm00439797_m1 |
|  | *Fmod* | Mm00491215_m1 |  |  | *Itga11* | Mm00723741_m1 |
|  | *Hspg2* | Mm01181173_g1 |  |  | *Itgb1* | Mm01253230_m1 |
|  | *Kera* | Mm00515230_m1 |  |  | *Itgb3* | Mm00443980_m1 |
|  | *Lum* | Mm01248292_m1 |  |  | *Itgb5* | Mm00439825_m1 |
|  | *Prg4* | Mm01284582_m1 |  |  | *Ptk2* | Mm00433209_m1 |
|  | *Vcan* | Mm01283063_m1 |  | **Cell Signaling** | *Bmp4* | Mm00432087_m1 |
| **Glycoproteins** | *Cd34* | Mm00519283_m1 |  |  | *Ctgf* | Mm01192933_g1 |
|  | *Cd44* | Mm01277161_m1 |  |  | *Egr1* | Mm00656724_m1 |
|  | *Comp* | Mm00489490_m1 |  |  | *Gdf5* | Mm00433564_m1 |
|  | *Eln* | Mm00514670_m1 |  |  | *Igf1* | Mm00439560_m1 |
|  | *Fbn1* | Mm00514908_m1 |  |  | *Ihh* | Mm00439613_m1 |
|  | *Fbn2* | Mm00515713_m1 |  |  | *Ltpb1* | Mm00498234_m1 |
|  | *Fn1* | Mm01256744_m1 |  |  | *Ltpb4* | Mm00723631_m1 |
|  | *Has1* | Mm03048195_m1 |  |  | *Mstn* | Mm01254559_m1 |
|  | *Matn2* | Mm00489626_m1 |  |  | *Mtor* | Mm00444968_m1 |
|  | *Mcam* | Mm00522397_m1 |  |  | *Pdgfa* | Mm01205760_m1 |
|  | *Pecam1* | Mm01242576_m1 |  |  | *Pdgfb* | Mm00440677_m1 |
|  | *Postn* | Mm01284919_m1 |  |  | *Pdgfrb* | Mm00435553_m1 |
|  | *Sparc* | Mm00486332_m1 |  |  | *Serpine1* | Mm00435858_m1 |
|  | *Spp1* | Mm00436767_m1 |  |  | *Tgfb1* | Mm01178820_m1 |
|  | *Thbs1* | Mm00449032_g1 |  |  | *Tgfb2* | Mm00436955_m1 |
|  | *Thbs2* | Mm01279240_m1 |  |  | *Tgfb3* | Mm00436960_m1 |
|  | *Thbs3* | Mm00449802_m1 |  |  | *Tgfbr2* | Mm03024091_m1 |
|  | *Thbs4* | Mm03003598_s1 |  |  | *Vegfb* | Mm00442102_m1 |
|  | *Tnc* | Mm00495662_m1 |  |  | *Yap1* | Mm01143263_m1 |
|  | *Tnmd* | Mm00491594_m1 |  | **Cell Markers** | *Acta2* | Mm00725412_s1 |
|  | *Tnxb* | Mm00466624_m1 |  |  | *Adgre1* | Mm00802529_m1 |
| **Cell Cycle** | *Mki67* | Mm01278617_m1 |  |  | *Mkx* | Mm00617017_m1 |
|  | *Pcna* | Mm00448100_g1 |  |  | *Scx* | Mm01205675_m1 |
| **Housekeepers** | *Abl1* | Mm00802029_m1 |  |  | *Sox9* | Mm00448840_m1 |
|  | *Rn18s* | Mm03928990_g1 |  |  |  |  |
|  | *Rps17* | Mm01314921_g1 |  |  |  |  |
